# Ankle Push-Off Based Mathematical Model for Freezing of Gait in Parkinson's Disease

**DOI:** 10.3389/fbioe.2020.552635

**Published:** 2020-10-29

**Authors:** Midhun Parakkal Unni, Prathyush P. Menon, Mark R. Wilson, Krasimira Tsaneva-Atanasova

**Affiliations:** ^1^Department of Mathematics, College of Engineering Mathematics and Physical Sciences, University of Exeter, Exeter, United Kingdom; ^2^Sport & Health Sciences, University of Exeter, Exeter, United Kingdom; ^3^Department of Bioinformatics and Mathematical Modelling, Institute of Biophysics and Biomedical Engineering, Bulgarian Academy of Sciences, Sofia, Bulgaria; ^4^Living Systems Institute, University of Exeter, Exeter, United Kingdom; ^5^EPSRC Centre for Predictive Modelling in Healthcare, University of Exeter, Exeter, United Kingdom

**Keywords:** Parkinson's disease, gait, chaos, neuro-mechanical model, modeling

## Abstract

Freezing is an involuntary stopping of gait observed in late-stage Parkinson's disease (PD) patients. This is a highly debilitating symptom lacking a clear understanding of its causes. Walking in these patients is also associated with high variability, making both prediction of freezing and its understanding difficult. A neuromechanical model describes the motion of the mechanical (motor) aspects of the body under the action of neuromuscular forcing. In this work, a simplified neuromechanical model of gait is used to infer the causes for both the observed variability and freezing in PD. The mathematical model consists of the stance leg (during walking) modeled as a simple inverted pendulum acted upon by the ankle-push off forces from the trailing leg and pathological forces by the plantar-flexors of the stance leg. We model the effect on walking of the swing leg in the biped model and provide a rationale for using an inverted pendulum model. Freezing and irregular walking is demonstrated in the biped model as well as the inverted pendulum model. The inverted pendulum model is further studied semi-analytically to show the presence of horseshoe and chaos. While the plantar flexors of the swing leg push the center of mass (CoM) forward, the plantar flexors of the stance leg generate an opposing torque. Our study reveals that these opposing forces generated by the plantar flexors can induce freezing. Other gait abnormalities nearer to freezing such as a reduction in step length, and irregular walking patterns can also be explained by the model.

## 1. Introduction

Parkinson's disease results from the loss of neurons in the substantia nigra pars compacta of the basal ganglia (BG) (Davie, 2008), which has projections toward the motor, and cognitive areas (Albin et al., 1989; Alexander and Crutcher, 1990). Freezing of gait (FoG) is a motor disability in PD patients where subjects experience an “episodic absence or marked reduction of forwarding progression of the feet despite the intention to walk” (Nutt et al., 2011, p 734). This debilitating symptom occurs during the late-stage PD (Giladi et al., 2001) and is known to be very difficult to predict and control. The physiology of this symptom is not yet established conclusively, consisting of both neural and mechanical components. A set of correlations between the neural inputs (e.g., Dysfunction of Visuomotor and occipito-parietal pathways) and mechanical variables (e.g., gait pattern generation and automaticity) of PD-FoG have been studied (Heremans et al., 2013) but causality is not well-established. Apart from freezing, abnormal gait patterns in PD consists of high stride time variability with less reduction in stride length (Heremans et al., 2013). The relationship between these abnormalities and freezing is also not well-understood.

Gait has been studied fundamentally from two different perspectives. One that of robotics and control, and the second, biophysical. Mathematical models of passive gait have been studied extensively by several authors to understand their stability (e.g., Goswami et al., 1996; Manchester et al., 2011; Dai and Tedrake, 2013; Sadeghian and Barkhordari, 2020), and the effect of external conditions such as ramps (McGeer, 1990) and bifurcations has been investigated (e.g., Mahmoodi et al., 2013; Iqbal et al., 2014; Fathizadeh et al., 2018, 2019; Znegui et al., 2020). Impulsive dissipation at heel strike is studied for a multidimensional biped model in (Ros et al., 2015). There are other approaches to motor control using optimal control which demands an arbitrary or learned cost-functionals (e.g., Flash and Hogan, 1985; Pekarek et al., 2007; Parakkal Unni et al., 2017). These models are not sufficient to understand human locomotion in PD patients as these papers have focused on the stability behaviors and control of robots. Some of these cost functional/error minimization based models, even though they assume the existence of such a cost, have the advantage of being useful for extracting parameters easily from the data (Delp et al., 2007; Wu et al., 2019). However, they do not address explicitly how the external inputs result in high variability and freezing observed in PD gait (Heremans et al., 2013).

On the other hand, a biophysical model proposed in Taga (1995) considers the interaction with the Central Pattern Generators (CPG). The aim of the model is primarily to demonstrate walking as a stable limit cycle that emerges from the dynamic interaction between neural oscillation originating in CPG and the pendulum oscillation of body linkages, rather than involuntary stoppage of gait and variability. CPG-based complex model, which depends on several parameters such as the strength of neural connections, the magnitude of the coefficients in the rhythmic force controller, and strength of sensory inputs, has its significance. However, one drawback of such CPG-based complex model is often the dependence on an excessive number of parameters as described above to be determined for achieving a desired locomotor pattern over a large search space. To identify and tune such parameters for attaining involuntary freezing and variability of gait behavior for a wider population of patients is rather an arduous trial-and-error or learning and optimization based task. Involuntary stoppage of gait and variability is the key detail that is necessary to show the model's ability to display PD walking behavior. The effect of opposing forces generated by the plantar flexors observed in PD, as reported in Nieuwboer et al. (2004), is yet another detail for understanding the PD gait.

The model by Muralidharan et al. (2014) successfully captures the neural dynamics of basal ganglia (BG) but does not focus on the mechanics. A model which combines the chaotic region of the Lorentz system with the passive dynamic walker by Montazeri Moghadam et al. (2018), adds chaos externally, which makes it less relevant biophysically. However, these authors have established a need for explaining the variable nature of PD walking. As chaos is known to be absent in the basal ganglia (BG) of a PD patient (Mandali et al., 2015) the neuro-mechanical interactions need to be studied to find out its underpinnings. Another way to look at gait biophysically is through the equilibrium point hypothesis (Feldman, 1986; Duan et al., 1997), which suggests movements are the result of active changes in the equilibrium state of the motor system. Torque length characteristics of the muscles can be changed by a neural controller to achieve motion. It has been shown that the muscles act synergistically to reduce the variability in the targeted action. For example, the uncontrolled manifold hypothesis by Latash et al. (2002) explains the variability in muscle recruitment as “good” and “bad” regions of variability, depending on whether they achieve the targeted action or not. The muscle recruitments which achieve targeted action are considered “good” regions of variability, whereas the muscle recruitments which does not achieve targeted action are considered as “bad” regions of variability. The Equilibrium point approach (Feldman, 1986) makes the electromyogram (EMG) activity implicit. Another limitation of the equilibrium point hypothesis is in its difficulty in testing. The empirical determination of invariant characteristics (Sainburg, 2015) such as torque-length characteristics (Feldman, 1986) is necessary for validating the equilibrium point hypothesis. A way in which it is achieved is by asking the subjects “not to intervene” (Feldman, 1986; Sainburg, 2015) while doing a task such as unloading and assuming this results in stabilization of central commands to muscles (Sainburg, 2015). But this assumption is not necessarily true as there could be involuntary responses. The neuromuscular system is over-actuated with redundancies, as it contains more actuators than the degree of freedom. Use of muscle synergies (Latash, 2010) in models is one way to address redundancies. These models assume co-activation of a set of muscles as motor primitives to address the redundancy associated with muscle activation (Aoi et al., 2019; Tamura et al., 2020). The idea of muscle synergy is still debated and is considered difficult to refute by any empirical evidence or falsify (Popper, 2002; Olszewski and Sandroni, 2011) by some authors (Tresch and Jarc, 2009).

There is a need for a model to explain the empirical observations in PD gait, such as the high coefficient of variability and freezing near narrow passages (Snijders et al., 2008). Such a model will help in understanding the essential aspects of neural and mechanical systems contributing to PD gait, also shedding light into the future experimentations required. In this work, the relationships between the high variability and freezing will be studied by deriving a set of forces acting on the stance leg. They phenomenologically represent the EMG (Electromyogram) signals and therefore the activity of the CPGs. Kinetics of both swing leg and stance leg will be studied to better understand their roles under the action of these forces.

**Figure 1:**
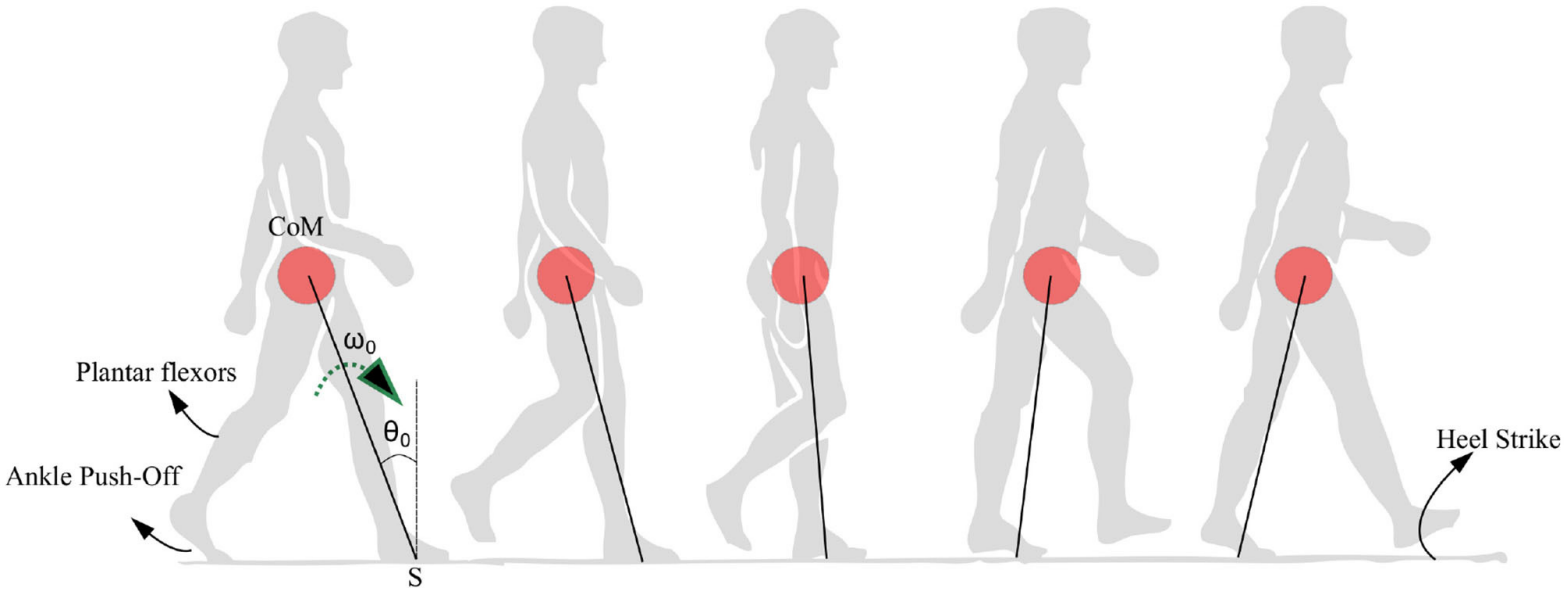
Anatomical representation of the stance phase from the ankle push-off to the heel strike is shown. The position of the center of mass (CoM) is shown as a red circle which is assumed to be rotating with respect to a pivot point S. The location of the plantar flexors, approximate region generating ankle push-off force and heel strike region are noted. The initial angle and angular velocity are represented by *θ*_0_ and *ω*_0_, respectively.

In summary, the model is built with two aims. The first aim is to explain the empirical observations that are seen in PD-Gait with a minimum number of variables. These include (1) a high coefficient of variation in PD subjects (Heremans et al., 2013), (2) a pattern of reduction of step lengths before freezing (Nutt et al., 2011), (3) the ability of sensory and visual cues to help reduce freezing (Rochester et al., 2005; Young et al., 2014; Amini et al., 2019), (4) the difficulty of freezing prediction, and (5) the occurrence of freezing near obstacles and narrow passages (Snijders et al., 2008). Secondly, the model aims to show the role of the swing leg as a supplier of ankle push of force as well as one that determines the time of heel strike. Hence, a bipedal model and a reduced low dimensional model resembling an inverted pendulum are studied upon the action of the ankle push-off force. The movement of the CoM under the action of the ankle push-off force is depicted in Figure 1. Hence, the hypothesis investigated in this study is that the variability and the motor symptoms associated with PD (Heremans et al., 2013) can be explained by the experimentally observed premature activation of plantar flexors observed in PD (Nieuwboer et al., 2004).

## 2. Materials and Methods—Modeling

### 2.1. Physiology

Walking is a complex process which involves the interaction of the brain, spinal cord and the musculoskeletal systems (Nutt et al., 2011). The typical gait cycle associated with walking involves “*stance*” and “*swing*” phases. The stance phase begins with a crucial heel strike phase, which is the initial contact that occurs instantaneously. As soon as the stance phase ends, the swing phase begins. The plantar flexor muscles of the trailing leg, supply energy to “push-off” the contra-lateral leading leg (Zelik and Adamczyk, 2016). Once the push-off occurs, the trailing leg enters the swing phase. The soleus and gastrocnemius muscles are the most notable plantar flexors, of which the significant role of the latter one in PD freezing/walking is established (Nieuwboer et al., 2004). Even though physiologically there is a non-linear relationship between the EMG signals and the torques generated (Genadry et al., 1988), a linear relationship can be assumed (Hof and Van Den Berg, 1977) between the envelope of the EMG (CPG firing) and the torques generated about the joint. Several other muscles are involved in walking, but the present study investigates only the effect of plantar flexors as these muscles supply most of the energy required for walking. In this work, the “freezing step” is defined as the step at which the legs do not have sufficient angular momentum to progress walking forward. When the physiology is modeled as an inverted pendulum-like system, the freezing results in backward motion of the stance leg. In a real-life scenario, this implies the patient either falls or stops movement. The remark 1 defines freezing and related terms used in this work.

Remark 1. *In this study, “freezing” or “freezing event” is defined as the condition where there is no forward motion of the stance leg. “Freezing episode” is defined as the events happening in the time interval between the heels strike phase the freezing event. Hence, the “start of the freeze” is defined to be at the heel strike phase after which a freezing event occurs.*

Here, we carry out a systematic stability analysis, including unstable regimes, of the model in contrast to the stable limit cycle behavior studied in robotics (Grizzle et al., 2001) and passive walking dynamics literature (McGeer, 1990). Even though the complex freezing behavior can be explained through several possible routes (Nutt et al., 2011) (some of them purely based on neural control) an attempt is made here to explain it in the simplest possible way and to understand the effect of neuromuscular inputs in generating unstable and chaotic walking behavior as observed in PD (Heremans et al., 2013).

### 2.2. Dynamics of Walking

The dynamics of walking involves the coordinated action of neural input and muscles of the limbs. It consists in a continuous movement of the limbs as well as state reset at heel strike resulting in discrete dynamics (Sinnet et al., 2011). Plantar flexors of the swing leg supply the necessary torque to push the CoM forward. During walking, the CoM is supported by leg in stance phase for the majority of the time (80-90%) as the double support phase is approximately 10-20% of the overall gait cycle (Wahde and Pettersson, 2002; Kharb et al., 2011). The motion of the CoM with single support under the action of the plantar flexors is modeled in this work. The heel strike is modeled using discrete dynamics.

Traditionally the dynamics of walking is often modeled as biped model (Taga, 1995). In this section, a simplified biped model is presented. Further, a reduced, low dimensional, *inverted pendulum system* is considered as a special case relevant to PD. It is assumed that CoM is at the tip of the pendulum, and the links and the swing leg are massless. Therefore, the model generates the motion of the CoM of the human body. Running and jumping gaits are not considered in this model as the links are assumed to be rigid. It is also assumed that sufficient friction exists to avoid any slip. The angular displacements are assumed to be small enough (< 0.5 rad.) (Usherwood, 2005; Ranavolo et al., 2011; Polese et al., 2012) to allow for first/second-order approximations during the stance phase. Kane's method (Kane and Levinson, 1985) is used to derive the equations of motion (EoM). Kane's dynamical equation is of the form ${\bar{F}}_{r}+{\bar{F}}_{r}^{*}=0$, where ${\bar{F}}_{r}$ and ${\bar{F}}_{r}^{*}$ represents generalized active forces and generalized inertia forces, respectively, as described in Kane and Levinson (1985) (chapter 6, page 159). The equations in the form necessary for simulation is obtained using python libraries, the details of which are given in Gede et al. (2013).

**Figure 2:**
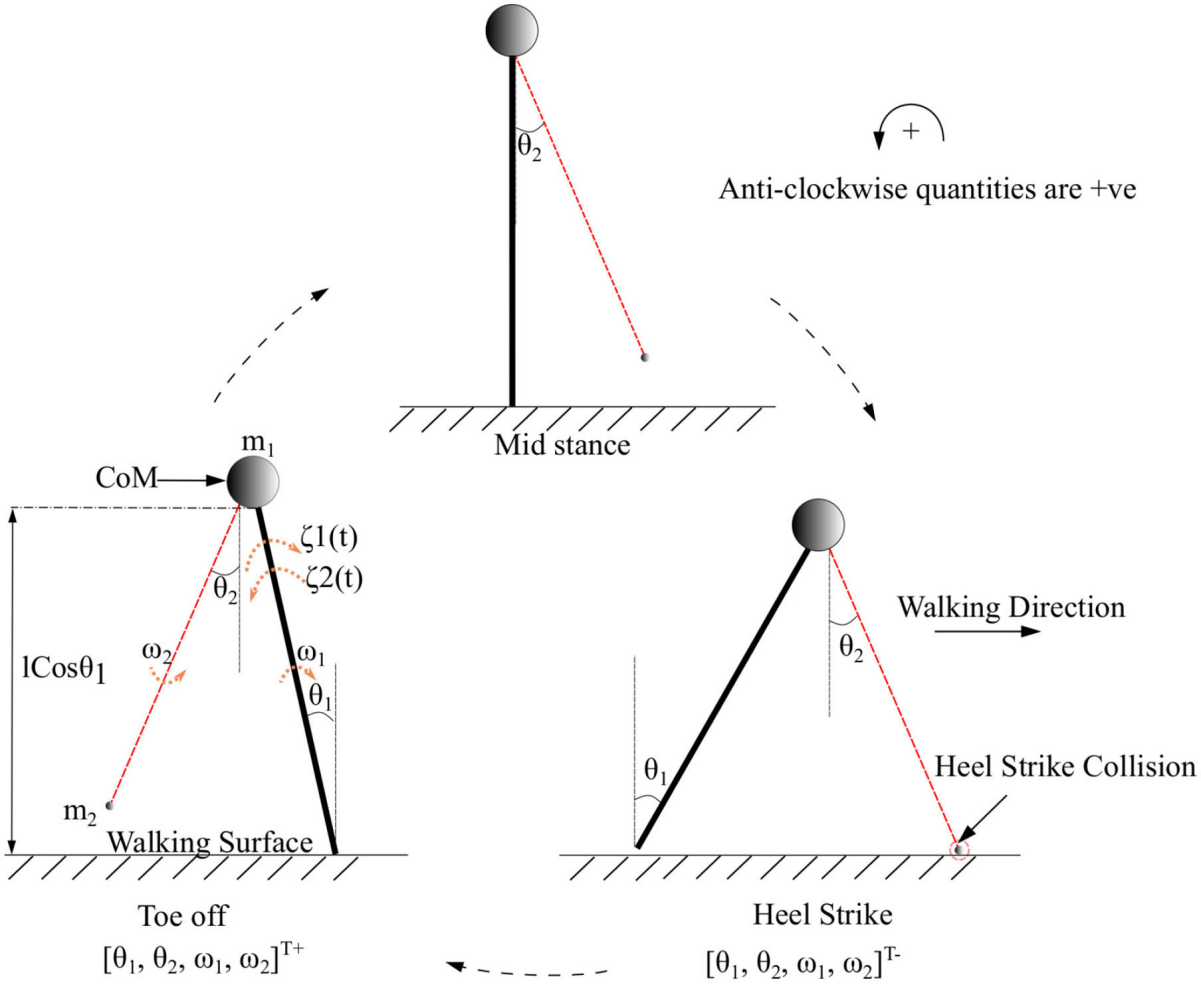
The toe-off, mid stance, and heel strike instances of the two connected links of the biped with its CoM while walking in the forward direction. CoM indicated as a circle represents a point mass at the tip of the pendulum. The swing leg is indicated in red. The point of collision during a heel strike instance is circled in red. The terms *θ*_1_ and *θ*_2_ are the angles that the stance and swing legs subtend w.r.t. the vertical (in the inertial reference frame), respectively. The quantities *ω*_1_, *ω*_2_ and *m*_1_, *m*_2_ are the corresponding angular velocities and masses of the body and swing leg, respectively. The torques that are acting on the stance leg are indicated as ζ_1_(*t*) (Ankle push-off force) and ζ_2_(*t*) (Torque due to the activation of the plantar flexors of the stance leg). States immediately before and after heel strike is indicated with “-” and “+” superscripts, respectively.

Symbols *m*_1_, *m*_2_ represent the mass of the body and swing leg, respectively, as shown in Figure 2. The length of both the legs is represented by *l*. The variables associated with the system are the components of the vector $x={\left[\theta_{1},\;\theta_{2},\;\omega_{1},\;\omega_{2}\right]}^{T}$ which are the angles and angular velocities (w.r.t inertial frame for stance leg and w.r.t. stance leg frame for swing leg) as indicated in Figure 2. There are two types of angular velocities. The one which corresponds to the rotation of the rigid body with respect to its center of rotation is called spin angular velocity and one which corresponds to the revolution of a point with respect to an origin is called orbital angular velocity. In this work, spin angular velocities with respect to the center of rotation of the rigid links are considered as they rotate about the center of rotation.

Hybrid systems (Lunze and Lamnabhi-Lagarrigue, 2009) are a class of dynamical systems, which exhibit both continuous states and discrete mode dynamics often associated with events such as resets, jumps, and switching. The continuous behavior is typically governed by a system of differential equations (similar to Equation 1) and the discrete part is governed by a vector-valued function (similar to Equation 2) (Lunze and Lamnabhi-Lagarrigue, 2009)(chapter 1). The transition between the discrete and continuous governing equations is determined by the state of the system in the overall phase space. The dynamics in this work is governed by the general hybrid dynamical system of the form,

(1) $$\begin{array}{l} \dot{x}=q\left(x\right),x^{-}\notin S \end{array}$$

(2) $$\begin{array}{l} x^{+}=\Delta \left(x^{-}\right),x^{-}\in S \end{array}$$

where,

(3) $$\begin{array}{l} S:=\left\{x\in \chi |g_{reset}\left(x\right)=0\right\} \end{array}$$

where, *q*(*x*), Δ(*x*^−^), and *g*_*reset*_(*x*) are continuous vector valued functions of *x*. In the absence of external torques acting on the leg, the term *q*(*x*) is,

(4) $$q(x)=\left[\begin{array}{c} \omega_{1}\\ \omega_{2}\\ \frac{1}{2l(m_{1}+m_{2}{\text{sin }}^{2}(\theta_{2}))}(2gm_{1}\text{sin }(\theta_{1})-gm_{2}\text{sin }(\theta_{1}+2\theta_{2})\\ +gm_{2}\text{sin }(\theta_{1})+lm_{2}\omega_{1}^{2}\text{sin }(2\theta_{2})-2lm_{2}\omega_{2}^{2}\text{sin }(\theta_{2}))\\ -\frac{\text{sin }(\theta_{2})}{l(m_{1}+m_{2}{\text{sin }}^{2}(\theta_{2}))}(gm_{1}\text{cos }(\theta_{1})+gm_{2}\text{cos }(\theta_{1})\\ -l\omega_{1}^{2}(m_{1}+m_{2})+lm_{2}\omega_{2}^{2}\text{cos }(\theta_{2})) \end{array}\right]$$

The function, Δ(.) is the reset map, χ ⊂ ℝ^4^ the state space, and *g*_*reset*_(.) is the function that defines the heel strike. The set “*S*” defines a surface where the heel strikes the ground and the states change abruptly according to the reset map. The *x*^−^ and *x*^+^ indicate the states immediately before and after the heel strike, respectively. The functions Δ(.) and *g*_*reset*_(.) are described in sequel.

As the body mass is considerably larger than the mass of the leg, the case where *m*_2_ goes to 0 has only been considered. Further, small-angle approximation leads to the following equation for ${\dot{\omega }}_{1}$ and ${\dot{\omega }}_{2}$

(5) $$\begin{array}{l} {\dot{\omega }}_{1}=g\frac{\theta_{1}}{l} \end{array}$$

(6) $${\dot{\omega }}_{2}=-\frac{(g-l\omega_{1}^{2})\theta_{2}}{l}$$

The ankle push-off forces of the stance leg supply majority of the energy needed to propel the leg forward. When this neuromuscular forcing Γ(*t*) is added to the stance leg, the Equation (5) becomes,

(7) $$\begin{array}{l} {\dot{\omega }}_{1}:=g\frac{\theta_{1}}{l}+\Gamma \left(t\right) \end{array}$$

The forcing term Γ(*t*) is derived in the following section.

### 2.3. Derivation of the Forcing Terms

**Figure 3:**
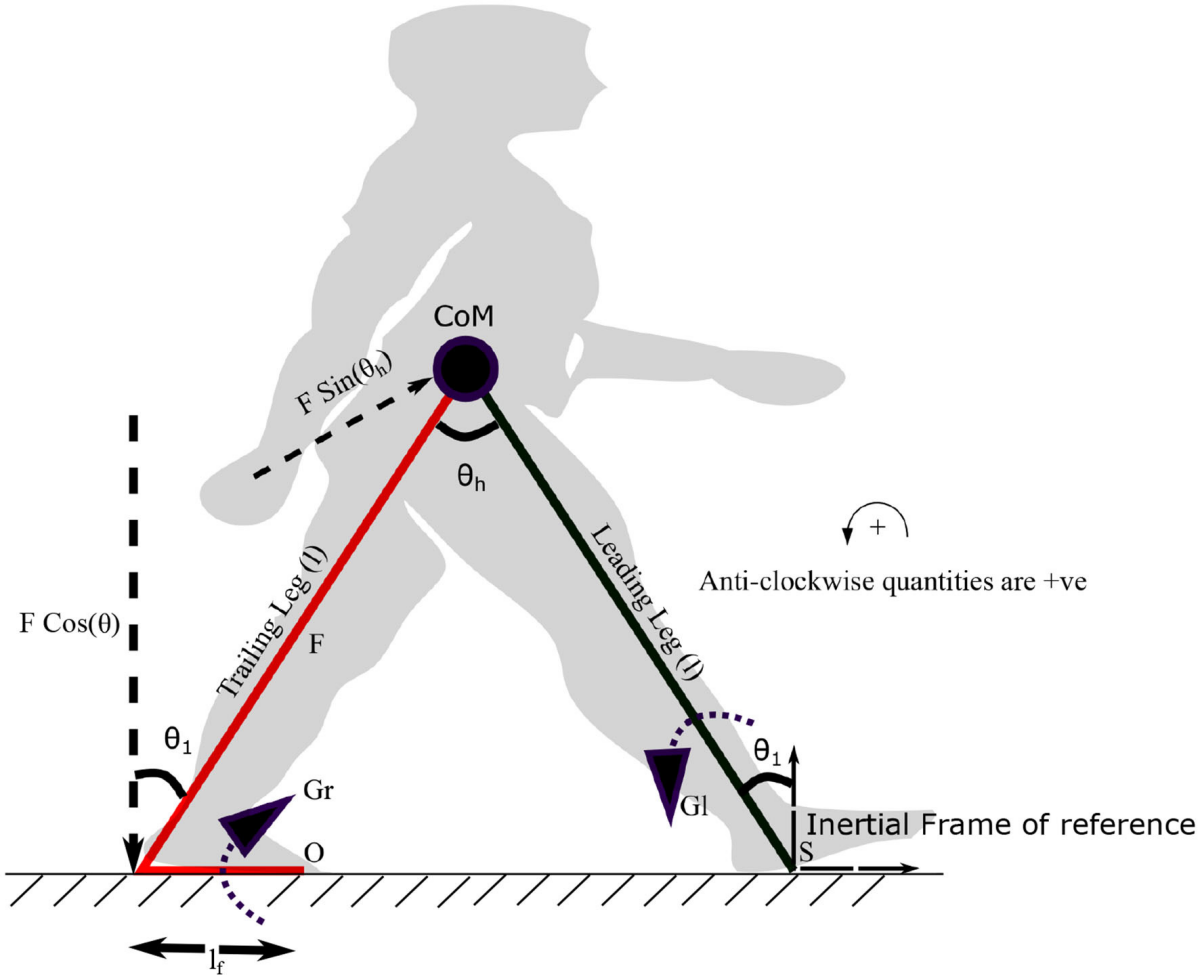
The diagram visualizes the forces and moments on leading and trailing leg that enable the movement of the center of mass (CoM) forward. Symbols S and O indicate the points on leading and trailing leg about which the torques *G*_*l*_ and *G*_*r*_ are applied. *G*_*r*_ is the torque generated by the plantar flexors of the trailing leg, which results in a force *F* (ankle push-off) acting on the leading leg, about the point “S.” The distance between the pivot point to the point of action of the force is *l*_*f*_. The plantar flexors of the leading leg generate a torque *G*_*l*_ in the leading leg, in the opposite direction. The angle the leading leg subtends with the vertical axis at S is *θ*_1_. *θ*_*h*_ represents the hip angle. The moments are balanced about “S” to get the equations of motion (EoM). An approximate position of the starting stance phase is shown in the background in gray color.

The torques acting on the stance leg are derived in this section to generate the neuromuscular forcing term Γ(*t*) for the stance leg. Torque produced by the plantar flexors on the trailing leg is defined as *G*_*r*_(*t*) and that on the leading leg as *G*_*l*_(*t*). These torques are assumed to be linearly related to the envelop of the EMG signals which are positive functions of time (Nieuwboer et al., 2004). The torque *G*_*r*_(*t*) generates the ankle push off force *F*(*t*) and is assumed to be in phase with the heel strike. In the proposed model, the force *F*(*t*) and the torque *G*_*l*_(*t*) are assumed to be

(8) $$\begin{array}{l} G_{l}\left(t\right):=\tau_{l}\left(\text{sin}\left(2\pi f_{r_{1}}t+\phi \right)+1\right) \end{array}$$

(9) $$\begin{array}{l} F\left(t\right):=\tau_{r}\left(\text{sin}\left(2\pi f_{r_{2}}t\right)+1\right) \end{array}$$

where τ_*l*_ and τ_*r*_ are constants. The variables, *f*_*r*_1__, *f*_*r*_2__ and ϕ represent frequencies and the phase difference between torques on the leading and trailing leg, respectively. Both frequencies are assumed to be unity. The ankle push off torque acting on the leading leg (in stance phase) can be calculated using the free body diagram shown in Figure 3. The pivot points of the trailing leg and leading leg are “O” and “S,” respectively. Trailing leg and leading leg subtends the same angle *θ*_1_ w.r.t. the normal to the ground as the trailing leg and leading leg together with the ground is assumed to form an isosceles triangle. By balancing the moments about the point “S” in Figure 3 yields,

(10) $$\begin{array}{l} I{\dot{\omega }}_{1}=G_{l}\left(t\right)-lF\left(t\right)\text{sin}\left(\theta_{h}\right)+mgl\text{ sin}\left(\theta_{1}\right) \end{array}$$

When angle *θ*_1_ is small (sin(θ) ≈ θ) and since $I=m_{1}l^{2}$, Equation (10) is rewritten as,

(11) $$\begin{array}{l} m_{1}l^{2}{\dot{\omega }}_{1}=G_{l}\left(t\right)-lF\left(t\right)\text{sin}\left(\theta_{h}\right)+m_{1}gl\theta_{1} \end{array}$$

Substituting *F*(*t*) and *G*_*l*_(*t*) from Equation (8) and (9) in Equation (11), one obtains,

(12) $$\begin{array}{l} m_{1}l^{2}{\dot{\omega }}_{1}=\tau_{l}\left(\text{sin}\left(2\pi f_{r_{1}}t+\phi \right)+1\right)-l\tau_{r}\left(\text{sin}\left(2\pi f_{r_{2}}t\right)+1\right)\text{sin}\left(\theta_{h}\right)\\ \begin{array}{l} \;+m_{1}gl\theta_{1} \end{array} \end{array}$$

Rearranging Equation (12), angular acceleration of the leading/stance leg is,

(13) $$\begin{array}{l} {\dot{\omega }}_{1}=\overset{\text{Plantar flexors of the leading leg}(=\zeta_{2}\left(t\right))}{\overset{︷}{\frac{\tau_{l}\left(\text{sin}\left(2\pi f_{r_{1}}t+\phi \right)+1\right)}{l^{2}m_{1}}}} \end{array}$$

(14) $$\begin{array}{l} -\overset{\text{Ankle push off from trailing leg}(=\zeta_{1}\left(t\right))}{\overset{︷}{\frac{\tau_{r}l\text{ sin}\left(\theta_{h}\right)\left(\text{sin}\left(2\pi f_{r_{2}}t\right)+1\right)}{l^{2}m_{1}}}}+\overset{Gravity}{\overset{︷}{\frac{g\theta_{1}}{l}}}\\ \begin{array}{l} \;:=\frac{\zeta_{2}\left(t\right)-\zeta_{1}\left(t\right)}{m_{1}l^{2}}+\frac{g\theta_{1}}{l} \end{array} \end{array}$$

where $\Gamma \left(t\right):=\frac{\left(-\zeta_{1}\left(t\right)+\zeta_{2}\left(t\right)\right)}{m_{1}l^{2}}$ (as shown in Figure 2) is the time varying neuromuscular forcing.

The initial velocity of the swing leg is assumed to be constant in every step. As the mass *m*_2_ is assumed to be zero, the corresponding angular momentum is also equal to zero. Therefore, the angular velocity of the stance leg is reset to conserve its angular momentum and the initial angular velocity of the swing leg after reset is assumed to be a positive constant to account for the impulse during the ankle push-off. This is a valid assumption as the definition of freezing in this work is independent of the swing leg movement. A reset is carried out when *θ*_1_ + *θ*_2_ = 0 and *θ*_1_ < 0. Using Equations (6) and (14), the equations of motion of a biped can be written as in Equations (1)–(2), where the functions *q*(*x*), Δ(*x*), *g*_*reset*_(*x*) and the set *S* are written as follows,

(15) $$\begin{array}{l} q\left(x\right):=\left[\begin{array}{c} \omega_{1}\\ \omega_{2}\\ \frac{\zeta_{2}\left(t\right)-\zeta_{1}\left(t\right)}{m_{1}l^{2}}+\frac{g\theta_{1}}{l}\\ -\frac{\left(g-l\omega_{1}^{2}\right)\theta_{2}}{l} \end{array}\right] \end{array}$$

(16) $$\begin{array}{l} \;S:=\{{\left[\begin{array}{c} \theta_{1},\;\theta_{2},\;\omega_{1},\;\omega_{2} \end{array}\right]}^{T}\in \chi \;|\;g_{reset}\left({\left[\begin{array}{c} \theta_{1},\;\theta_{2},\;\omega_{1},\;\omega_{2} \end{array}\right]}^{T}\right)\\ \;=0\wedge \theta_{1}<0\},\\ g_{reset}(.):=\theta_{1}+\theta_{2},\\ \;\Delta (.):=\left[-\theta_{1},\;-\theta_{2},\;\omega_{1}\text{ cos}(\theta_{h}),\;\omega_{2}^{0}\right] \end{array}$$

with initial conditions $\omega_{1}\left(0\right)=\omega_{1}^{0},\;\theta_{1}\left(0\right)=\theta_{1}^{0},\;\omega_{2}\left(0\right)=\omega_{2}^{0},\;\theta_{2}\left(0\right)=\theta_{2}^{0}$.

Remark 2. *It may be noted that the torque generated by the plantar flexors is assumed to act about the point “O” as the pivot point. Balancing the moments due to the ankle push off force, *F*(*t*) and *G*_*r*_(*t*) about the point O, the ankle push-off force can be determined in terms of *G*_*r*_(*t*) as,*

(17) $$\begin{array}{l} F\left(t\right)=\frac{G_{r}\left(t\right)}{l_{f}\text{cos}\left(\theta_{1}\right)} \end{array}$$

Here, the distance, *l*_*f*_ is taken between the heel to the pivot point on the foot for calculating the moments, as shown in Figure 3. Therefore, implicitly, the following assumption has been made while prescribing *F*(*t*).

(18) $$\begin{array}{l} \frac{G_{r}\left(t\right)}{l_{f}\text{ cos}\left(\theta_{1}\right)}:=\tau_{r}\left(\text{sin}\left(2\pi f_{r_{2}}t\right)+1\right) \end{array}$$

### 2.4. Rationale for Using a Low Dimensional Model for Analysis

The angular velocity of the stance leg contributes directly to the angular acceleration of the swing leg. A higher absolute angular velocity of the stance leg leads to lower acceleration of the swing leg. However, the dynamics of the stance leg in Equation (14) is uncoupled from the dynamics of the swing leg, and hence resembles the dynamics of an “*inverted pendulum system*.” It may be noted that the term “$l\omega_{1}^{2}$” can be approximated to a constant as in the physiological range of low angular velocities (especially in PD patients) $g>>l\omega_{1}^{2}$ (typically quantity $l\omega_{1}^{2}=0.448\text{ m}.{\text{rad}}^{2}.s^{-2}=0.7\times 0.8^{2}$ is of order “0” while *g* = 9.8 m.s^−2^ is of order 1). This results in a condition where the swing leg acts independently to the stance leg, effectively determining the step length. Therefore, an inverted pendulum walking model for PD subjects is valid when constant step length is assumed. Figure 1 depicts the physical rationale behind the use of an inverted pendulum model. The constant step length assumption is general enough to explain the variability in stepping as this leads to variability in stepping angular velocities rather than step lengths. In summary, in the following sections we present analysis of the stance phase walking model in light of the PD walking behavior at a constant step length. Physiologically, the hip applies torques on the swing leg and controls its initial angular velocity. The hip torques acting on the swing is not relevant in propelling the CoM forward, as most of the torque required for that is supplied by the ankle (Zelik and Adamczyk, 2016). Therefore, an assumption made on the swing leg angular velocity will not affect the applicability of the model to the freezing problem as freezing is related to the inability of the legs to propel the CoM forward in the case of walking. Hence, swing leg angular velocity is reset to $\omega_{2}^{0}$ in every step. Furthermore, the low dimensional model helps to avoid making any assumptions on the initial angular velocity of the swing leg $\omega_{2}^{0}$ as it doesn't involve a swing leg.

## 3. Analysis of the Reduced System

When considered independently of the swing leg, the dynamics has states corresponding only to the stance leg, i.e., $x={\left[\theta_{1},\;\omega_{1}\right]}^{T}$. The terms defining the Equation (1)–(2) for the inverted pendulum case are given below.

(19) $$\begin{array}{l} q\left(.\right):=\left[\begin{array}{c} \omega_{1}\\ \frac{\zeta_{2}\left(t\right)-\zeta_{1}\left(t\right)}{m_{1}l^{2}}+\frac{g\theta_{1}}{l} \end{array}\right] \end{array}$$

(20) $$\begin{array}{l} S\;:=\left\{{\left[\begin{array}{c} \theta_{1},\omega_{1} \end{array}\right]}^{T}\in \chi |g_{reset}\left({\left[\begin{array}{c} \theta_{1},\omega_{1} \end{array}\right]}^{T}\right)=0\right\} \end{array}$$

(21) $$\begin{array}{l} g_{reset}\left(.\right):=\theta_{1}-\theta_{reset} \end{array}$$

(22) $$\begin{array}{l} \Delta \left(.\right):={\left[\begin{array}{c} -\theta_{1},\omega_{1}\text{cos}\left(\theta_{h}\right) \end{array}\right]}^{T} \end{array}$$

**Figure 4:**
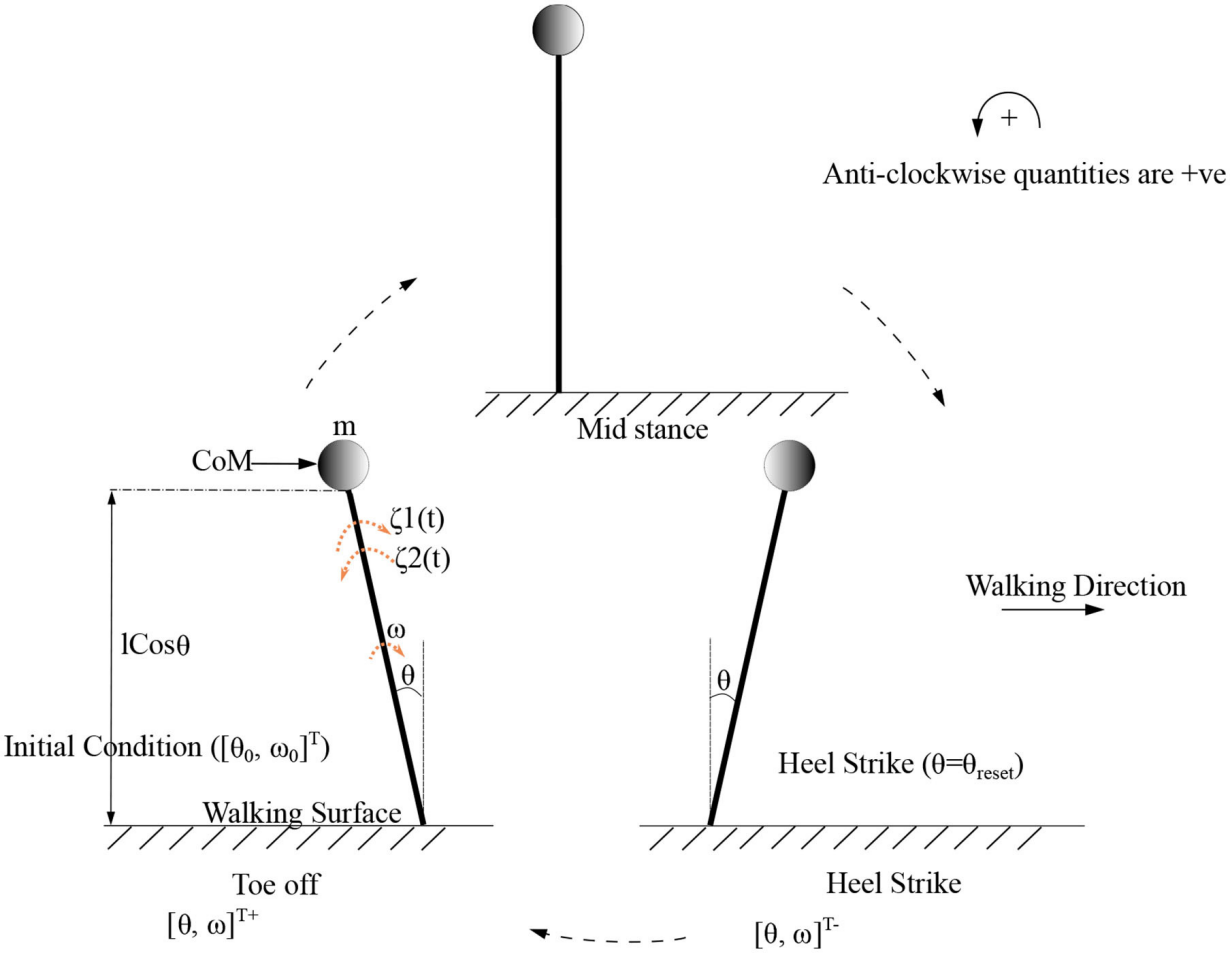
Gait cycle for the low dimensional (*inverted pendulum system*) system is shown. Terms ${\left[\theta_{0},\omega_{0}\right]}^{T}$ and ${\left[\theta_{0},\omega_{0}\right]}^{T-}$ indicate the initial and final angular position and velocity of the leading/stance leg, respectively, at the beginning and end of the stance phase. *θ*_*reset*_ is the angular displacement at which the angle is reset. The CoM (center of mass) which is assumed to be acting as a point mass at the tip of the inverted pendulum is shown as a circle. States before and after heel strike is indicated with “-” and “+” superscripts, respectively.

As the inverted pendulum model is analyzed independently, *θ*_1_, *m*_1_, $\theta_{1}^{0}$ and $\omega_{1}^{0}$ will be referred here as θ, *m*, *θ*_0_ and *ω*_0_, respectively. These equations are solved to produce the motion trajectory during the stance phase of the stepping cycle. The sequence of model evolution is depicted in Figure 4, with the beginning and end of the stance positions, initial angular position (*θ*_0_), initial angular velocity (*ω*_0_) and the angle at reset (*θ*_*reset*_). Step length is defined to be equal to |*θ*_*reset*_| where |.| denotes the absolute value.

### 3.1. Gait Cycle

The proposed model considers only the “*stance”* phase of the gait cycle. Therefore, “gait cycle” in this study has been defined as the process, where the model states evolve from an initial condition of a step (“double support phase”) until the reset condition (where the heel of the swing leg is assumed to collide with the ground or “heel strike condition”) is met and the initial condition of the next step is computed. Here, the state of the system moves through three different states (beginning of the “*stance”* (double support), end of the “*stance”* (before collision of the contra-lateral leg), heel strike (after collision of the contra-lateral leg) whose notations are given below (Equation 23) ^1^^,^^2^.

(23) $$\begin{array}{l} {\left[\theta_{0},\omega_{0}\right]}^{T}\mapsto \underset{\text{Before collision}}{\underset{︸}{{\left[\theta_{0}^{-},\omega_{0}^{-}\right]}^{T}}}\mapsto \underset{\text{After collision}}{\underset{︸}{{\left[\theta_{0}^{+},\omega_{0}^{+}\right]}^{T}}}={\left[\theta_{1},\omega_{1}\right]}^{T} \end{array}$$

Here ${\left[\theta_{0},\;\omega_{0}\right]}^{T}$, ${\left[\theta_{0}^{-},\;\omega_{0}^{-}\right]}^{T}$, ${\left[\theta_{0}^{+},\;\omega_{0}^{+}\right]}^{T}$ correspond to the states at the initiation of the step, states at the end of the flow “immediately” before collision, and states “immediately” after collision, respectively. The states immediately after collision form the initial condition for the next step ${\left[\theta_{1},\;\omega_{1}\right]}^{T}$. The superscripts (“-,” “+”) need not indicate the relative sizes of the states but the chronological order in which they appear, that is “-” superscript represent before collision variables and “+” represents after collision. But it should be noted that the transformation from ${\left[\theta_{0}^{-},\;\omega_{0}^{-}\right]}^{T}$ to ${\left[\theta_{0}^{+},\;\omega_{0}^{+}\right]}^{T}$ happens instantaneously in the model. Counterclockwise angles are defined as positive. In a typical walking simulation this results in θ^−^ < 0 < θ^+^. That is, the stance phase ends at a negative value for the angle and resets to a positive value before beginning the next stance phase.

Subscripts (indicating the step number) will be dropped from before and after collision state symbols when the step number is not relevant for the derivation (Equation 24). The same superscript will be used while referring to other parameters which change during collision ^3^.

The states [θ, ω]^*T*^ evolve as a function of time except at the collision point, where the same time point maps to two different state values. ^4^

(24) $$\begin{array}{l} {\left[\theta_{n},\omega_{n}\right]}^{T}\mapsto \underset{\text{Before collision}}{\underset{︸}{{\left[\theta^{-},\omega^{-}\right]}^{T}}}\mapsto \underset{\text{After collision}}{\underset{︸}{{\left[\theta^{+},\omega^{+}\right]}^{T}}}={\left[\theta_{n+1},\omega_{n+1}\right]}^{T} \end{array}$$

### 3.2. Heel Strike Condition

A heel strike is defined as the state at which the swing leg (trailing leg) collides with the ground. This is modeled using an appropriate reset condition. At heel strike, both the angle and angular velocity are reset from the “before collision” to “after collision” state as described in Equation (24). The collision of the swing leg (trailing leg) at heel strike is modeled to be inelastic with angular momentum conserved. Therefore, the magnitudes of the angular momentum about the point of collision after and before collision are equated in the following way to generate the transition rule for angular velocity

(25) $$\begin{array}{l} lm\left(v^{+}\text{ sin}\left(90\right)\right)=lm\left(v^{-}\text{ sin}\left(90+\theta_{h}\right)\right) \end{array}$$

(26) $$\begin{array}{l} lm\left(l\omega^{+}\right)1=lm\left(l\omega^{-}\right)\text{ cos}\left(\theta_{h}\right) \end{array}$$

(27) $$\begin{array}{l} \omega^{+}=\omega^{-}\text{ cos}\left(\theta_{h}\right) \end{array}$$

at the *n*^*th*^ iteration(step)

(28) $$\begin{array}{l} \omega_{n+1}=\omega_{n}^{+}=\omega_{n}^{-}\text{ cos}\left(\theta_{h}\right) \end{array}$$

where *θ*_*h*_ is the hip angle. The angle, on the other hand, will be reset from θ^−^ to −θ^−^. This results in the following transition rules at $\theta^{-}=\theta_{reset}$

(29) $$\begin{array}{l} \omega_{n}^{+}=\omega_{n}^{-}\text{ cos}\left(\theta_{h}\right) \end{array}$$

(30) $$\begin{array}{l} \theta_{n}^{+}=-\theta_{n}^{-} \end{array}$$

Rearranging we obtain Δ(.) as

(31) $$\begin{array}{l} \Delta \left({\left[\begin{array}{c} \theta ,\dot{\theta } \end{array}\right]}^{T-}\right)={\left[\begin{array}{c} -\theta ,\dot{\theta }\text{ cos}\left(\theta_{h}\right) \end{array}\right]}^{T}\;. \end{array}$$

### 3.3. Analytical and Numerical Solution of the Equations of Motion

The differential equation Equation (1), was solved using the definition of the vector field given in Equation (19) analytically to obtain the flows given below.

(32) $$\begin{array}{l} \theta \left(t\right)=f_{\theta }\left(t,\omega_{0},\theta_{0}\right):=N_{1}/D_{1}\\ \omega \left(t\right)=f_{\omega }\left(t,\omega_{0},\theta_{0}\right)=\frac{d}{dt}\left(f_{\theta }\right):=N_{2}/D_{2} \end{array}$$

where *N*_1_, *N*_2_, *D*_1_, *D*_2_ are given in Supplementary Section 1. The analytical solution is intended to be used for the bifurcation analysis as numerical solutions may not always be able to detect the chaotic behavior (Lozi, 2013). The analytical solution will therefore be used to generate the discrete map governing the motion in the following sections. A numerical solution of the Equations (1)–(2) using definitions given in Equations (19)–(22) with the appropriate reset conditions (in the physiological range) are solved to show the freezing behavior and dynamics in the phase plane. PD subjects freeze intermittently, and the amount of time the subject walks until the freeze is an important measure to quantify the transient walking behavior. A simulation for a 10 s- window is carried out for different values of the parameters τ_*l*_ and τ_*r*_ (for a constant initial condition). The total time for which transient walking behavior occurred is computed numerically as a function of the parameters τ_*l*_ and τ_*r*_. Numerical methods are also used in solving boundary value problems to gain further insights into the system as given in the remark 3.

Remark 3. *The parameters τ_*l*_ and τ_*r*_ determines the amount of energy supplied to the system apart from gravity. To understand how they influence the kinetic energy of the system, the difference in speed between the initial and final states are compared for the boundary value problem with boundary conditions *θ*_0_ = 0 rad. and *θ*_0.5_ = −0.1 rad. with definitions given in Equations (19)–(22) unchanged. Here the boundary conditions are chosen from the physiological range.*

The quantities τ_*l*_, τ_*r*_, ω, ϕ, *θ*_*reset*_ and step length has units N m, N, rad. s^−1^, rad., rad., and rad., respectively, when not specified.

### 3.4. Derivation of a Map to Describe Successive Stance Phases

The evolution of the flow (given by Equation 32) is terminated when the swing leg meets the ground. In other words, when there is sufficient energy in the system for forward motion, there exists a “reset time” *T*(*θ*_0_, *ω*_0_) such that *f*_θ_(*T*(*θ*_0_, *ω*_0_), *ω*_0_, *θ*_0_) = *A*_*reset*_(*θ*_0_). Here, *A*_*reset*_(.) is a function of the joint angle and the ground that determines the angle of the stance leg while the foot strikes the ground. The arguments associated with the reset time *T*(*θ*_0_, *ω*_0_) will be dropped and will be referred to as *T* from here on. Accounting the transition rules in Equation (29) for reset and conservation of angular momentum,

one defines^5^

(33) $$\begin{array}{l} \theta_{1}=\theta^{+}\left(T\right):=-f_{\theta }\left(T,\omega_{0},\theta_{0}\right) \end{array}$$

(34) $$\begin{array}{l} \omega_{1}=\omega^{+}\left(T\right):=f_{\omega }\left(T,\omega_{0},\theta_{0}\right)\text{ cos}\left(\theta_{h}\right) \end{array}$$

Following an induction hypothesis, for an arbitrary initial condition (*θ*_*n*_, *ω*_*n*_) the map is

(35) $$\begin{array}{l} \theta_{n+1}=-f_{\theta }\left(T,\omega_{n},\theta_{n}\right) \end{array}$$

(36) $$\begin{array}{l} \omega_{n+1}=f_{\omega }\left(T,\omega_{n},\theta_{n}\right)\text{ cos}\left(\theta_{h}\right) \end{array}$$

The following definitions are made to make the notations compact for further analysis

(37) $$\begin{array}{l} \theta_{n+1}={\tilde{f}}_{\theta }\left(T,\omega_{n},\theta_{n}\right):=-f_{\theta }\left(T,\omega_{n},\theta_{n}\right) \end{array}$$

(38) $$\begin{array}{l} \omega_{n+1}={\tilde{f}}_{\omega }\left(T,\omega_{n},\theta_{n}\right):=f_{\omega }\left(T,\omega_{n},\theta_{n}\right)\text{ cos}\left(\theta_{h}\right) \end{array}$$

where ${\tilde{f}}_{\omega }\left(T,\;\omega_{n},\;\theta_{n},\;\phi \right)$ and ${\tilde{f}}_{\theta }\left(T,\;\omega_{n},\;\theta_{n},\;\phi \right)$ are *T* parametrized family of maps for (*ω*_*n*_, *θ*_*n*_) ↦ (*ω*_*n*+1_, *θ*_*n*+1_).

To investigate the condition of same step lengths and to generate a 1D map for further evaluation, *A*_*reset*_(θ, *t*) is set to be *θ*_*reset*_. Here *θ*_*reset*_ is an arbitrary angle in the physiological range at which the swing leg meets the ground. Then for an intermediate step, (when there is sufficient energy to move forward) there exists a $\tilde{T}$ s.t. ${\tilde{f}}_{\theta }\left(\tilde{T}\left(\theta_{n},\;\omega_{n}\right),\;\omega_{n},\;\theta_{n}\right)=\theta_{n}=-\theta_{reset}$. When there is not enough energy and therefore momentum to move forward, the model behavior is defined as freezing.

To find the $\tilde{T}$ at which *θ*_*n*_ maps to itself the following minimization problem is solved ^6^ using Newton's method (Wolfram Research Inc., 2019). This detects implicitly the time at which the swing leg collides with the ground.

(39) $$\begin{array}{l} \tilde{T}\left(\theta_{n},\omega_{n}\right):=\underset{T}{\text{arg min}}\left({\tilde{f}}_{\theta }\left(T,\omega_{n},\theta_{n},\phi \right)-\theta_{n}\right) \end{array}$$

Substituting $\tilde{T}$ from Equation (39) in Equation (38) the following map is obtained.

$$\begin{array}{l} \omega_{n+1}={\tilde{f}}_{\omega }\left(\tilde{T}\left(\theta_{n},\omega_{n}\right),\omega_{n},\theta_{n}\right)\\ \;:=H\left(\omega_{n}\right)\text{ when }\theta_{n}=\theta_{0}\forall n\in \mathbb{N} \end{array}$$

When used as a 1D map,

(40) $$\begin{array}{l} \omega_{n+1}:={\tilde{f}}_{\omega }\left(\omega_{n}\right) \end{array}$$

The argument $\tilde{T}$ in the function will be dropped from here-on. The function $\tilde{T}$ acts on the same input *ω*_*n*_ and *θ*_*n*_ = *θ*_0_. This map has been analyzed to show the freezing behavior and variabilities associated with PD walking. The map has been analyzed for a particular parameter value to show the presence of horseshoe in the Supplementary Section 2.

Remark 4. *Equations (1)–(2) represent a general hybrid system. When the hybrid bipedal system's solution is sought these equations are solved using the definitions given in Equations (15)–(16); and, Equations (19)–(22) are used for hybrid inverted pendulum system.*

## 4. Results

Numerical simulation of the PD gait and associated freezing behavior is described in this section. The change in the angular velocity from negative to zero is a property of any solution containing freezing by definition. Typically in this model, the angular velocity changes to a positive value under the action of gravity during a freeze. The effect of variation of the parameters τ_*l*_, τ_*r*_, ϕ, *θ*_*reset*_ are also investigated. The work aims to show that, the two opposing torques modeled to be generated from the plantar flexors could elicit freezing and chaotic behavior. The ability of these torques to generate freezing behavior has been shown first in a simplified biped model described using Equations (15)–(16), and then in the inverted pendulum model generated by Equations (19)–(22). As argued previously the inverted pendulum dynamics sufficiently captures the PD walking scenario. The results are presented in the sequel to support this hypothesis. Also, walking is the process of moving the CoM by pushing the stance leg forward, and the inverted pendulum model helps to study the effect of the stance leg independent of other variables. A range of values for the constants τ_*l*_, τ_*r*_, and ϕ have been analyzed, such that the trend in behavior is clear to understand. The range in which the behavior of the map ${\tilde{f}}_{\omega }$ changes the number of periodic orbits from “0” to “more than one” in lower absolute value of angular velocity conditions, is given Table 1. Simulations are carried out to understand the behavior of the system over and above this range. But it may be noted that the maximum value of the torque for *l* = 0.6 m., *θ*_*reset*_ = −0.1 rad. is approximately 0.23|τ_*r*_| N m and 2|τ_*l*_| N m in forward and backward directions, respectively. Hence, in this case, forward pushing plantar flexors has to generate 8.7 times the “premature activation of plantar flexors” to nullify the effect if the phase is matched exactly. Physiologically the minimum value of these torques is zero and the maximum is subject-specific.

**Table 1:** Summary of qualitative behavior of the map.

**No**	**Parameter**	**Range simulated**	**Figure no**	**Consequence of increasing the parameter**
1	τ_*l*_	[0, 5]	Figure 10	Increased τ_*l*_ results in the appearance of period 1-2-3 and higher orbits. This results in freezing at lower absolute angular velocity conditions
2	ϕ	[-6.28, -1.28]	Figure 11	Increase in ϕ results in the period doubling bifurcations as described in the Figure 9. When everything else remains constant a variation in ϕ results in freezing and high variability in walking.
3	τ_*r*_	[30, 55]	Figure 12A	Increased τ_*r*_ results in disappearance of period 1-2-3 and higher orbits. This is one of the ways in which the patients get out of a freeze
4	*θ*_*reset*_	[0.05, 0.15]	Figure 12B	Increased step length results in freezing region change its location on the map, from low initial absolute angular velocity to a higher absolute angular velocity initial conditions.

### 4.1. Freezing in a Biped Model

**Figure 5:**
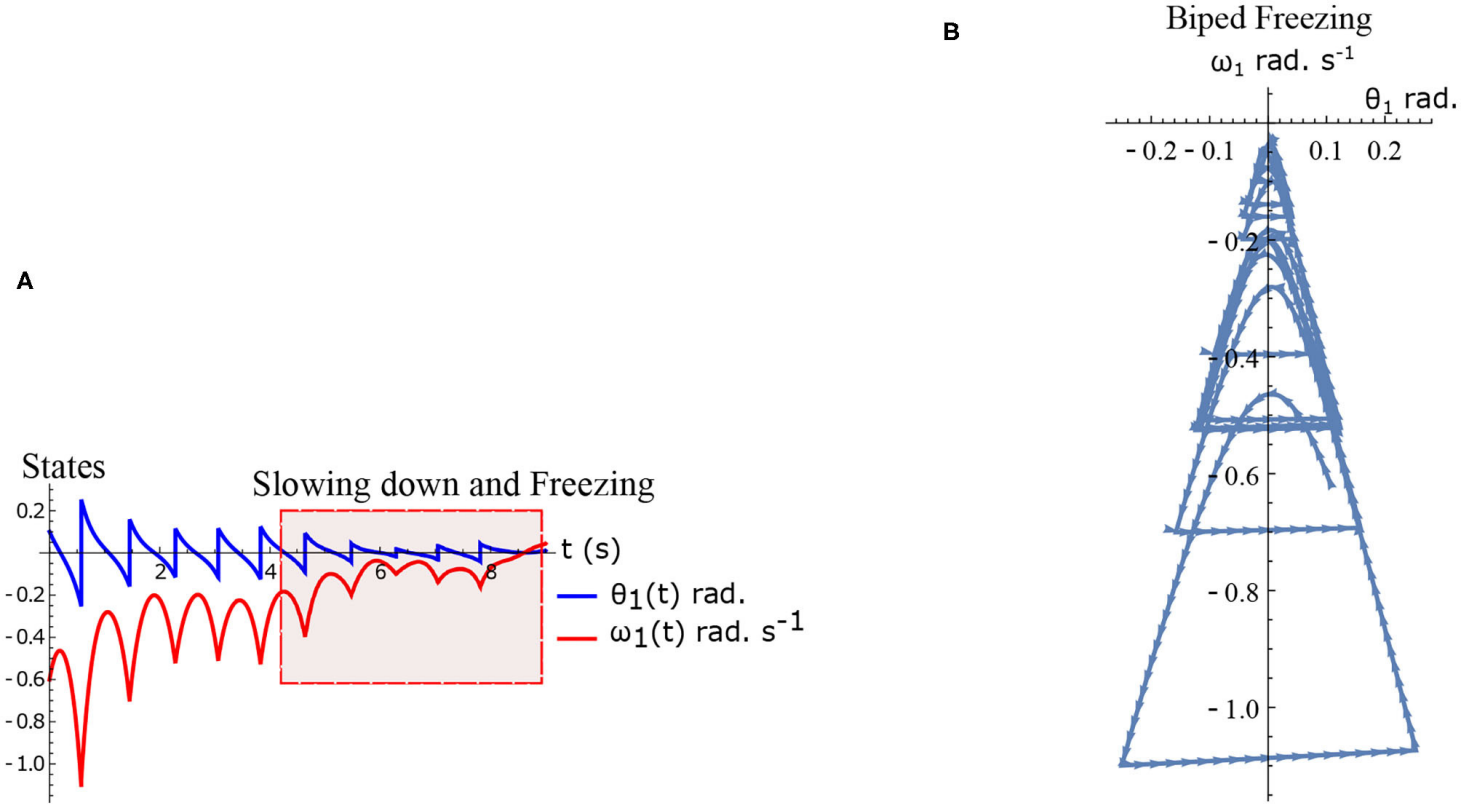
Results of numerical simulation of the biped during freezing. Parameters are chosen to be τ_*l*_ = 2.3 N m, τ_*r*_ = 15.74 N, ϕ = −π/2 rad. and initial conditions $\theta_{2}\left(0\right)=-0.1\;\text{rad}.,\;\theta_{1}\left(0\right)=0.1\;\text{rad}.,\;\omega_{2}\left(0\right)=2\;\text{rad}.s^{-1},\;\omega_{1}\left(0\right)=-0.6\;\text{rad}.s^{-1}$. The change in the *θ*_*reset*_ in every step and gradual reduction in *θ*_1_ nearer to a freezing event is evident. **(A)** Simulated time series of the states *θ*_1_ and *ω*_1_. Region of slowing down and freezing is highlighted **(B)** Shows numerical simulation of biped in the phase plane.

The hybrid system (Equations 1–2) defined by the Equations (15)–(16) are simulated numerically and the results are shown in Figures 5A,B. The figure shows normal walking for the first few steps and then freezing afterwards (highlighted). The gradual reduction in step length observed experimentally prior to freezing (Nutt et al., 2011) is also observed in the model.

**Figure 6:**
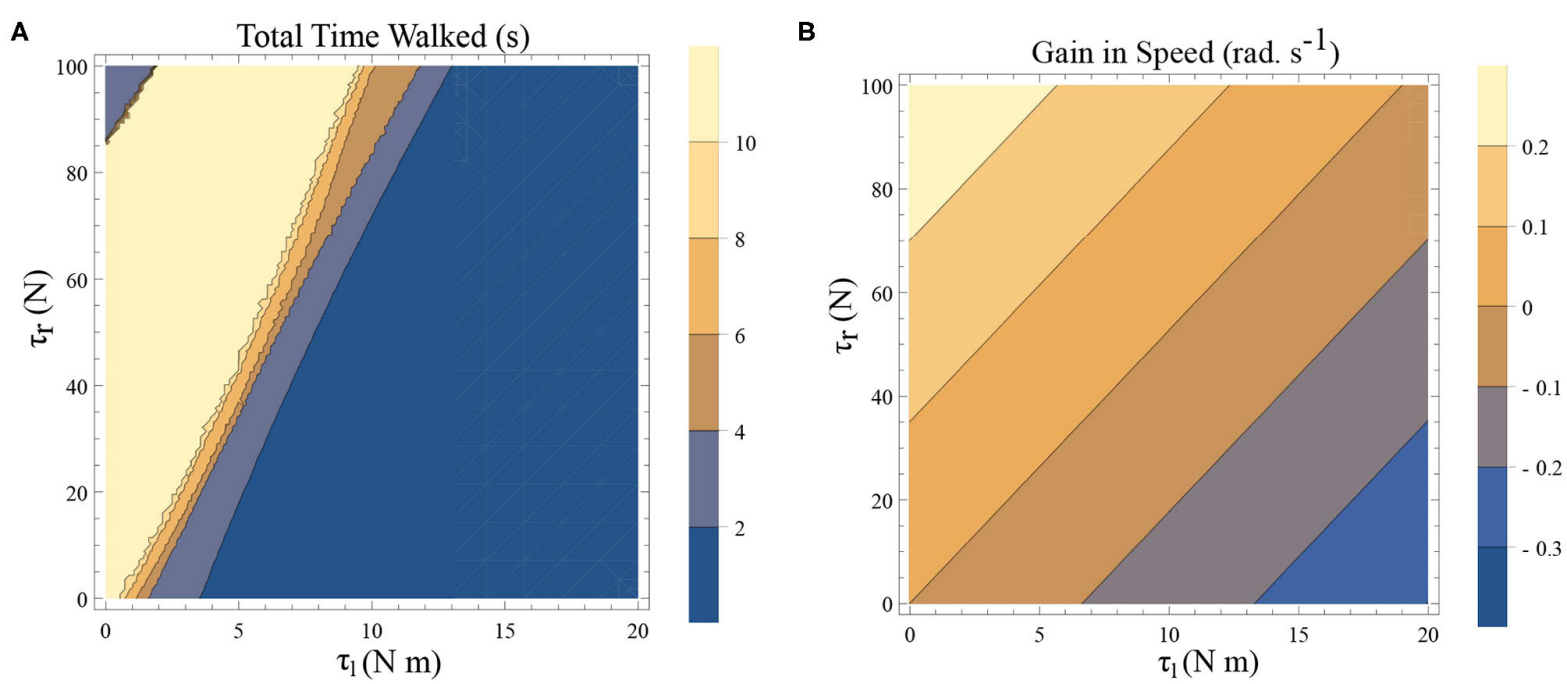
**(A)** A simulation was carried out for a time window of 10 s, and the total time walked before freezing was computed. This time is plotted as a function of τ_*l*_ and τ_*r*_. The initial conditions are set to be $\theta_{2}\left(0\right)=-0.1\;\text{rad}.,\;\theta_{1}\left(0\right)=0.1\;\text{rad}.,\;\omega_{2}\left(0\right)=1\;\text{rad}.s^{-1},\;\omega_{1}\left(0\right)=-0.6\;\text{rad}.s^{-1}$ and ϕ = −π/2 rad. The yellow region forms the optimal region of the parameters τ_*l*_, τ_*r*_ where walking is achieved. **(B)** Contour plot of the difference in speed(|ω(0)| − |ω(0.5)|) as a function of τ_*l*_ and τ_*r*_ determined by solving a BVP numerically with $\theta_{0}^{0}=0$ and $\theta_{1}^{0.5}=-0.1\;\text{rad}.$ for the biped model with the other initial conditions being $\theta_{2}^{0}=-0.1\;\text{rad}.,\;\omega_{2}^{0}=1$ (and ϕ = −π/2 rad.). This illustrates a decrease in the speed and therefore kinetic energy when there is an increase in τ_*l*_ and a decrease in τ_*r*_.

There are two dissipative forces in this model; they are, the opposing torques due to the plantar flexors and the dissipation at the heel strike. Long-range walking will be achieved when the speed gain in every step compensate these two effects. The dissipative effect of the heel strike can't be controlled by the neuromuscular system, but the effect of plantar flexors can. Figures 6A,B illustrates the effect of the plantar flexors in this regard.

A simulation was carried out for a 10 s window and the time difference between, the start of the simulation and the time of the last heel strike before the freezing event (as defined in remark 1), has been computed. This is shown as a function of the parameters τ_*l*_ and τ_*r*_ in Figure 6A. The blue shades indicate eventual freezing and shorter walking time and the yellow region is the safer non-freezing region. There is an intermediate region of parameter τ_*l*_ and τ_*r*_ in which the walking happens without freezing in the 10 s window. A higher value of τ_*l*_ necessitates a higher τ_*r*_ for walking. But a very high τ_*r*_ doesn't necessarily produce balanced walking as it can result in a lack of coordination between the swing leg and the stance leg. Although the initial value problem (IVP) in Figure 6A and boundary value problem (BVP) in Figure 6B can't be directly compared, they show analogous qualitative results. That is, to achieve the same speed gain [or kinetic energy (KE) gain] a higher τ_*l*_ demands a higher τ_*r*_. A key aspect of PD freezing is, therefore, the inability of the two plantar flexors to coordinate to produce the required energy. From an energy point of view, the role of the swing leg is mainly in the generation of ankle push of force. In the following sections, the dynamics of the stance leg is studied independently using an inverted pendulum model, reducing the role of the swing only as a supplier of the ankle push-off force.

### 4.2. Freezing in an Inverted Pendulum Model

**Figure 7:**
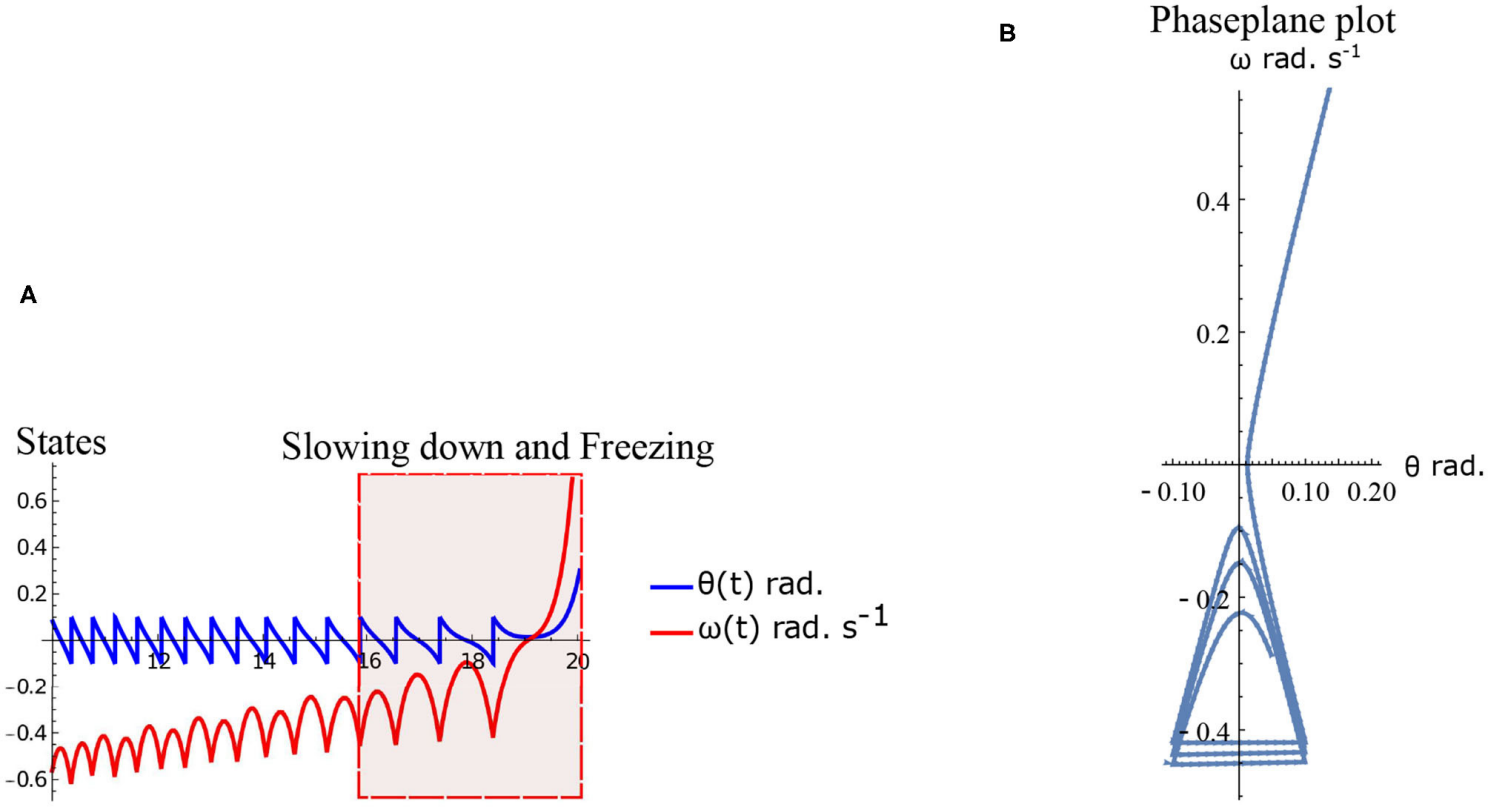
Simulation of the inverted pendulum dynamics for the parameter values $\phi =-\pi /2\;\text{rad}.,\;\omega_{0}=-1\;\text{rad}.s^{-1},\;\tau_{l}=2\;\text{N m},\;\tau_{r}=11\text{ N}$. Freezing occurs after 18 s. **(A)** States θ and ω as a function of time (10–20 s). Region of slowing down and freezing is highlighted. **(B)** Numerical simulation of the inverted pendulum states in the phase plane for 16–20 s.

**Figure 8:**
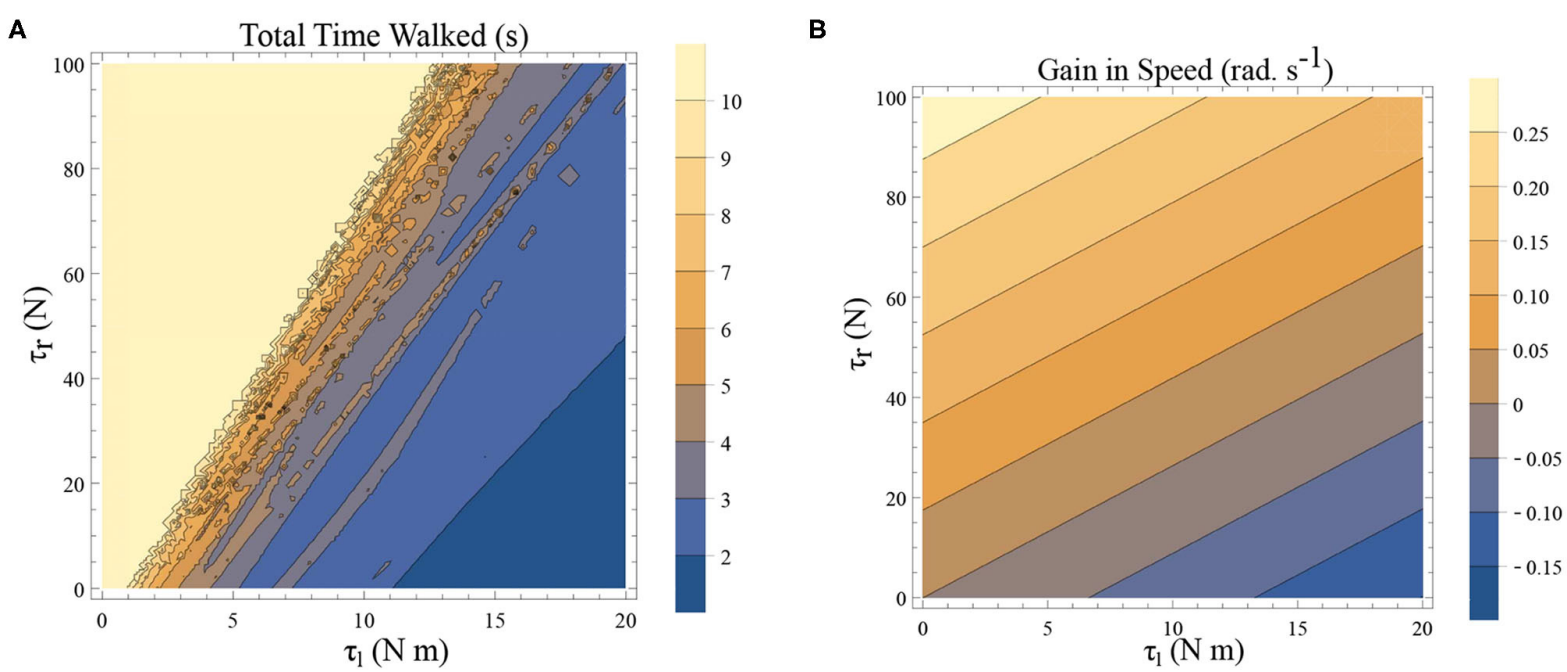
**(A)** A simulation was carried out for a time window of 10 s, and the total time of walking before freezing was computed. This time is plotted as a function of τ_*l*_ and τ_*r*_. The angle is reset when θ(*t*) = −0.1 rad. and ϕ = −π/2 rad. The colors indicate the duration of the walk (see the legend). **(B)** Contour plot of difference in speed (|ω(0)| − |ω(0.5)|) as a function of τ_*l*_ and τ_*r*_ determined by solving a BVP with *θ*_0_ = 0 rad. and *θ*_0.5_ = −0.1 rad. (and ϕ = −π/2 rad.) in Equation (1)–(2) using definitions in Equation (19)–(22). This illustrates a decrease in the speed and therefore KE when there is an increase in τ_*l*_ and a decrease in τ_*r*_.

Freezing is defined as the condition where there is no more forward motion of the leg. Numerical simulation of such a scenario in the inverted pendulum model is shown in Figure 7A where there is a freezing episode after 18 s. A gradual reduction in step length observed in the biped model translate to the increased time taken in making the final few steps before freezing. The simulation in the phase plane for the last three steps is shown in Figure 7B. The dissipative torques due to the opposing plantar flexors act in the same way in the case of the inverted pendulum model. Figures 8A,B illustrate this similarity, where, an increased τ_*r*_ generates higher speed gains and, an elevated τ_*l*_ results in lower speed gains and lower total walk times. This is because increasing parameter τ_*r*_ heightens the forward ankle push-off while larger τ_*l*_ amplifies the dissipative torque. A key difference between the inverted pendulum model and the biped model is that a higher τ_*r*_ will not result in an imbalance in the former as there is no swing leg in that model, while there is a lack of balance in the latter. The contour plot of the speed differences as a function of τ_*l*_ and τ_*r*_ is shown in Figure 8B. The figure shows that a higher value of τ_*l*_ and a lower value of τ_*r*_ result in negative speed gain (reduction in KE). Numerical simulation of the total time of the walk, defined as the difference between the time in which the first step is taken and the last step before freezing in 10 s is shown in Figure 8A. More than 9 s of walking is indicative of the fact that there is no freezing in that parameter range in that time frame. A higher τ_*r*_ and lower τ_*l*_ results in better walking performance as in the case of a biped. Therefore, energetically, PD related behavior that is of interest is analogous in the case of inverted pendulum and biped model. Therefore, the analytical solution of the inverted pendulum model is investigated further to understand the consequence of the change in parameters τ_*l*_, τ_*r*_ and ϕ. These parameters are controlled by the neural system while others such as mass of the body and length of the legs are not. The quantity *θ*_*reset*_ differentiates the inverted pendulum model from the biped model. Hence, the effect of this parameter is also studied.

### 4.3. Parameter Exploration of the Inverted Pendulum: Study of the Map ${\tilde{f}}_{\omega }$

**Figure 9:**
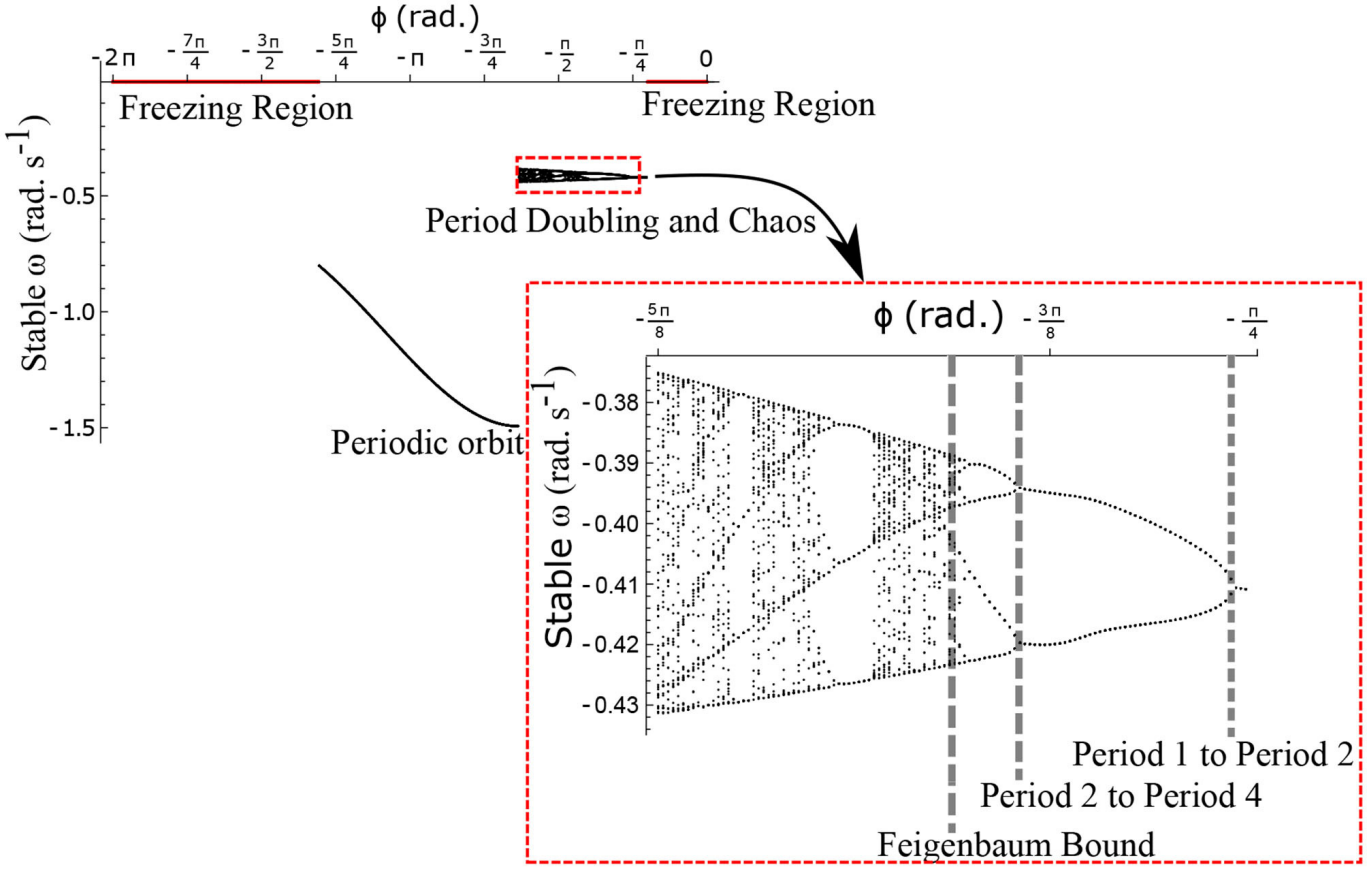
Stable ω is shown as a function of the parameter ϕ for $\omega_{0}=-0.433\;\text{rad}.s^{-1},\;\tau_{l}=5\;\text{N m},\;\tau_{r}=35\text{ N}$. The Feigenbaum bound was found to be at ϕ = −1.37 rad. where walking becomes fully chaotic. The period-doubling cascade has been highlighted and enlarged. This chaotic region forms only a small part of the overall parameter space of ϕ. This region is sandwiched between the walking and the freezing regions indicated in red.

**Figure 10:**
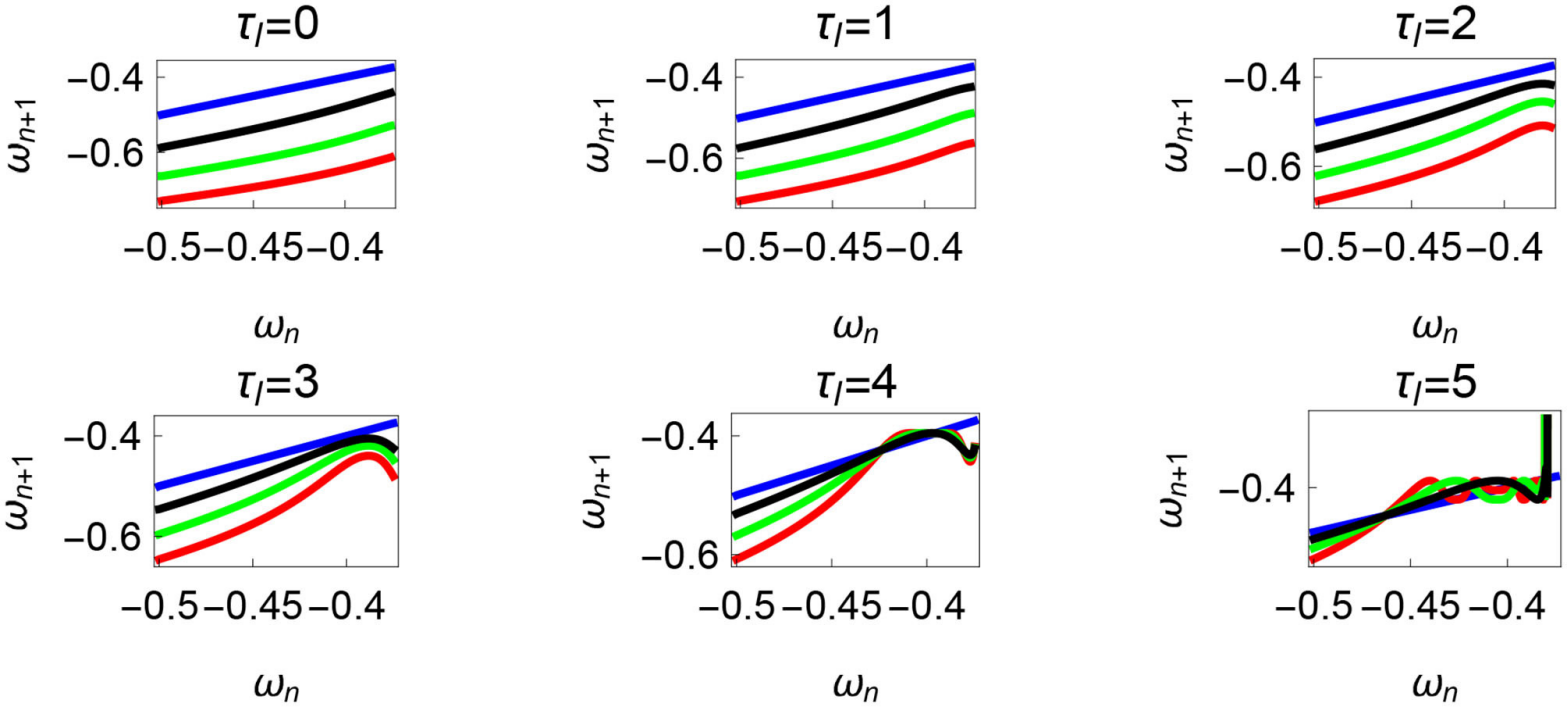
Maps obtained by varying the parameter τ_*l*_ and fixing τ_*r*_ = 35 N, ϕ = −1.57 rad., *θ*_*reset*_ = −0.1 rad. The black, green, and red curves represent ${\tilde{f}}_{\omega }^{1}$, ${\tilde{f}}_{\omega }^{2}$, ${\tilde{f}}_{\omega }^{3}$, respectively, and the *ω*_*n*_ = *ω*_*n*+1_ is shown in blue. The curves intersect the blue line at a higher absolute value of angular velocity forming an attractor, this is not shown in the figure. There are no periodic orbits for the low velocity regimes for τ_*l*_ = 0 − 2 N m but they appear afterwards. Units: τ_*l*_, τ_*r*_, ω, ϕ, *θ*_*reset*_ and step length has units N m, N, rad. s^−1^, rad., rad., and rad., respectively, when not specified.

**Figure 11:**
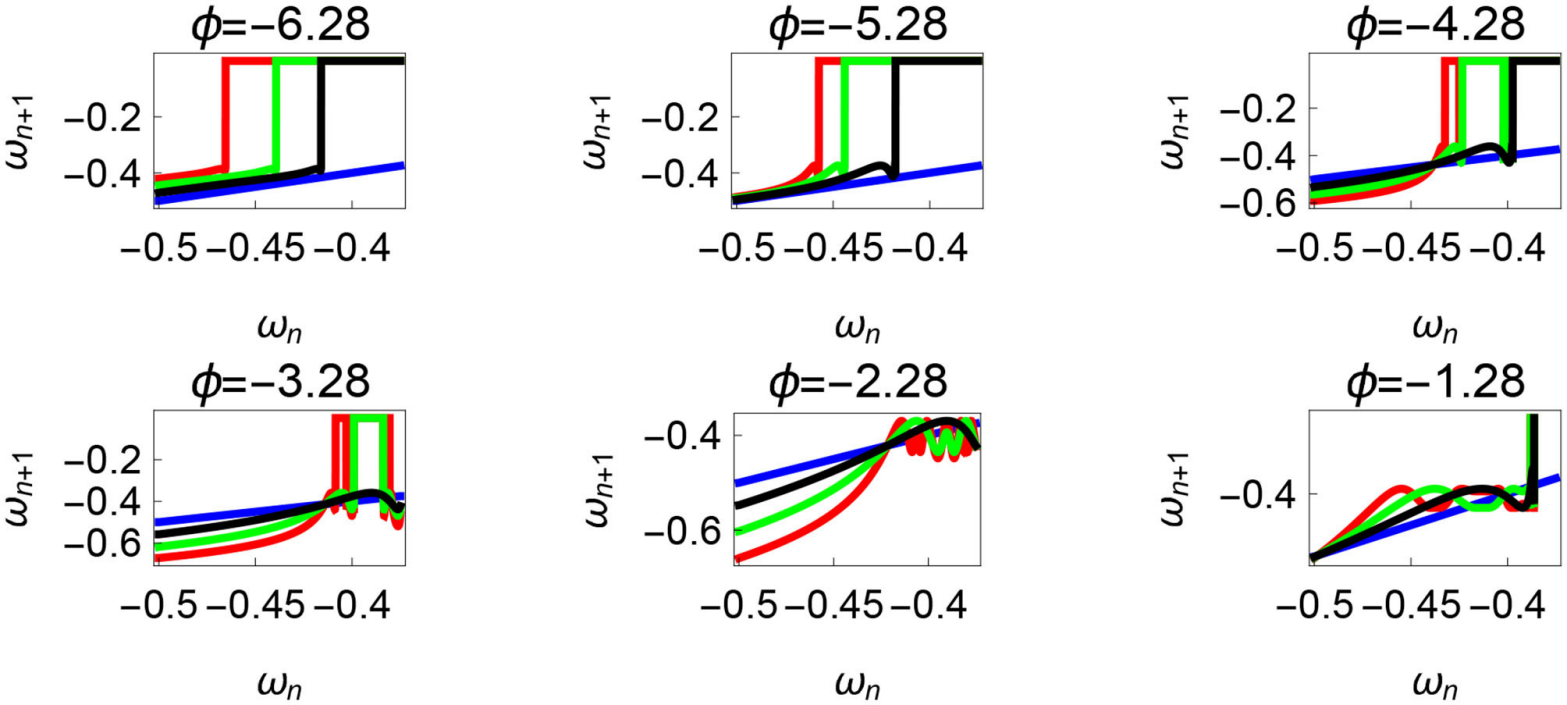
Varying the parameter ϕ and fixing τ_*r*_ = 35 N, τ_*l*_ = 5 N m, *θ*_*reset*_ = −0.1 rad. The black, green, and red curves represent ${\tilde{f}}_{\omega }^{1}$, ${\tilde{f}}_{\omega }^{2}$, ${\tilde{f}}_{\omega }^{3}$, respectively, and the *ω*_*n*_ = *ω*_*n*+1_ is shown in blue. The curves intersect the blue line at a higher absolute value of angular velocity forming an attractor, this is not shown in the figure. Creation of the periodic orbits and its coexistence is observed. Units: τ_*l*_, τ_*r*_, ω, ϕ, *θ*_*reset*_ and step length has units N m, N, rad. s^−1^, rad., rad., and rad., respectively, when not specified.

**Figure 12:**
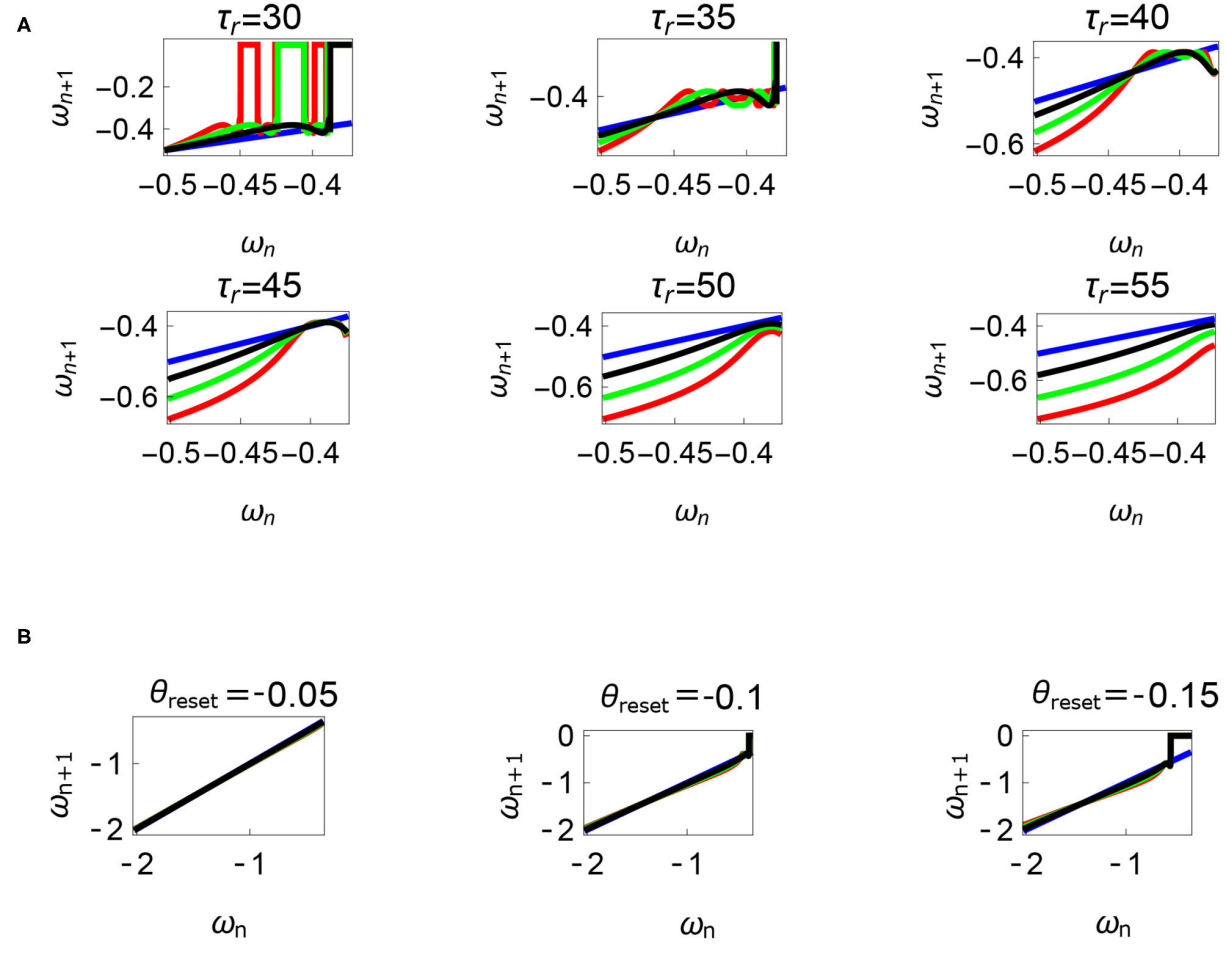
Variations of the parameters τ_*r*_ and *θ*_*reset*_ are depicted in Figures 12A,B, respectively. Units: τ_*l*_, τ_*r*_, ω, ϕ, *θ*_*reset*_ and step length has units N m, N, rad. s^−1^, rad., rad., and rad., respectively, when not specified. **(A)** Varying the parameter τ_*r*_ keeping τ_*l*_ = 5 N m, ϕ = −1.57 rad., *θ*_*reset*_ = −0.1 rad. fixed. The black, green, and red curves represent ${\tilde{f}}_{\omega }^{1}$, ${\tilde{f}}_{\omega }^{2}$, ${\tilde{f}}_{\omega }^{3}$, respectively, and the *ω*_*n*_ = *ω*_*n*+1_ is shown in blue. The curves intersect the blue line at a higher absolute value of angular velocity forming an attractor, this is not shown in the figure. Increasing τ_*r*_ has an analogous behavior as decreasing τ_*l*_. **(B)** Varying the parameter *θ*_*reset*_ keeping τ_*r*_ = 35 N, ϕ = −1.57 rad., τ_*l*_ = 0.5 N m. The black, green and red curves (overlapped) represent ${\tilde{f}}_{\omega }^{1}$, ${\tilde{f}}_{\omega }^{2}$, ${\tilde{f}}_{\omega }^{3}$, respectively, and the *ω*_*n*_ = *ω*_*n*+1_ is shown in blue. The curves intersect at a high absolute value of angular velocity. The unstable region moving to the higher absolute value of angular velocities (moving to the left) can be observed while |*θ*_*reset*_| is increased.

**Figure 13:**
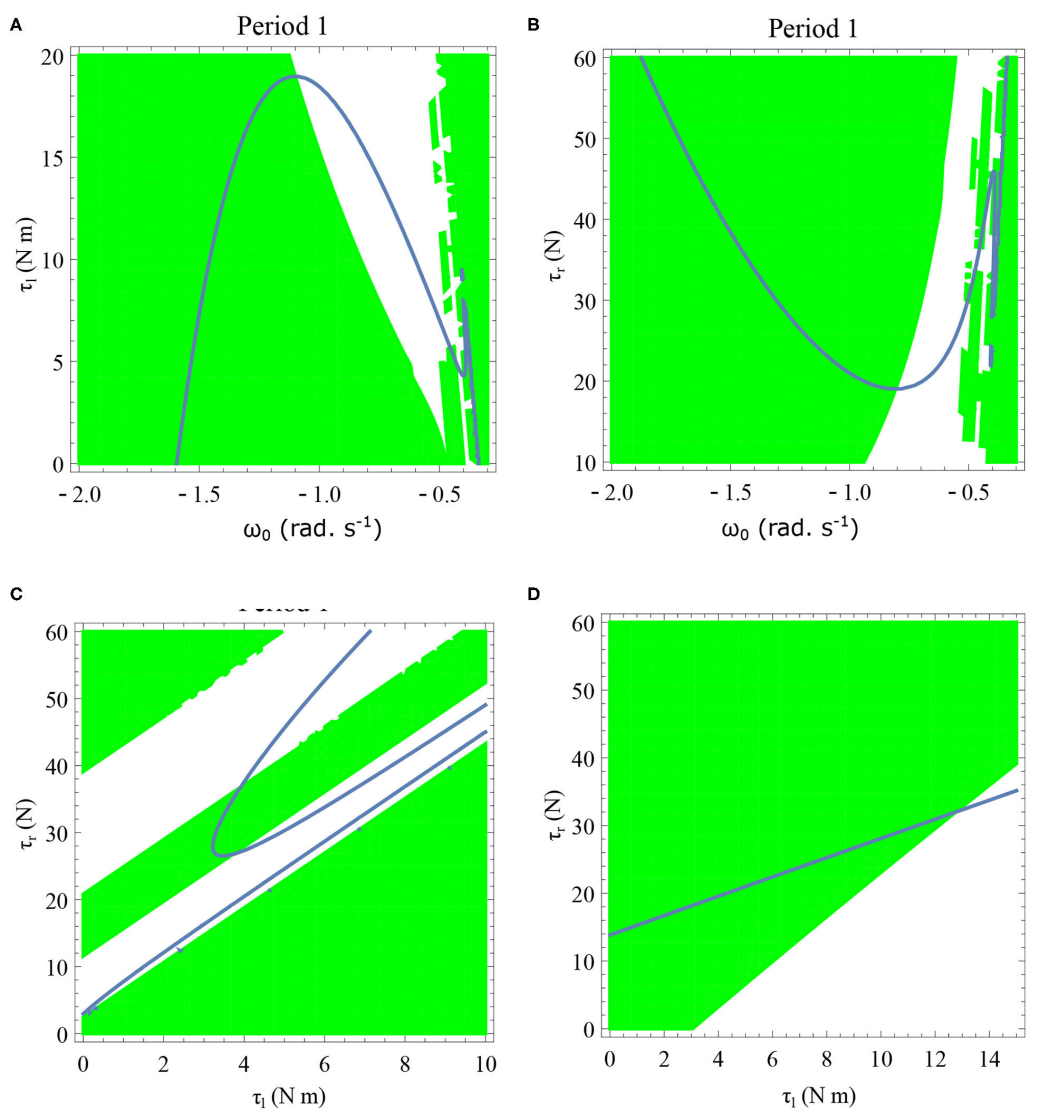
Period one orbits are shown by varying τ_*l*_ and τ_*r*_ for two different values of initial angular velocities in **(C,D)**. Period one orbits found by varying τ_*l*_ and *ω*_0_ is shown in **(A)**. Period one orbits found by varying τ_*r*_ and *ω*_0_ is shown in **(B)**. The parameter values used are given in the respective figures. The green region shows the stable region where ${\dot{\tilde{f}}}_{\omega }<1$. The stable periodic regions are the ones where the green region overlap the curves of the periodic orbits. Units: τ_*l*_, τ_*r*_, ω, ϕ, *θ*_*reset*_ and step length has units N m, N, rad. s^−1^, rad., rad., and rad., respectively, when not specified. **(A)** ϕ = −π/2 rad., τ_*r*_ = 40 N, *θ*_*reset*_ = −0.1 rad. **(B)** ϕ = −π/2 rad., τ_*l*_ = 5 N m, *θ*_*reset*_ = −0.1 rad. **(C)** ϕ = −π/2 rad., *ω*_0_ = −0.4 rad. s^−1^, *θ*_*reset*_ = −0.1 rad. **(D)** ϕ = −π/2 rad., *ω*_0_ = −1 rad. s^−1^, *θ*_*reset*_ = −0.1 rad.

The neural control on the muscles alters the magnitude and the phase of the control signals. Exploration of the parameters τ_*l*_, τ_*r*_, and ϕ, therefore, reflects the effect of the neural control on walking dynamics. One of the hypotheses that are investigated through the model is that of the generation of variability through the premature activation of the plantar flexors. We have quantified the phase difference of the “premature” activation using the parameter ϕ in the model. Figure 9 shows the bifurcation diagram of the parameter ϕ in the range 0 to −2π for constant values of τ_*l*_ and τ_*r*_. A period-doubling route to chaos can be observed when ϕ is varied between −5π/8 and −π/4. The map ${\tilde{f}}_{\omega }$ is iterated for 500 walking cycles and the last 50 walking cycles are used to compute the equilibrium points. The Feigenbaum bound is found to be at ϕ = −1.37 rad. at which walking becomes fully chaotic. This indicates that the premature activation (or lack of coordination between the muscles) can generate highly variable behavior in the system despite deterministic neural signals. The region of chaotic ϕ is sandwiched between the periodic orbits and freezing region. This suggests a higher variability in walking likely arising from a shift in ϕ (early activation of plantar flexors) must be treated with caution. Figure 9 shows the presence of chaos in the system for carefully selected parameter values. Its presence and stability are illustrated for other parameters values and initial conditions using a set of maps in Figures 10–12B and bifurcation diagrams in Figures 13A–D. A summary of the insights obtained from the maps are given in the Table 1. The presence of a period 3 orbit in a one-dimensional map is indicative of other periodic orbits and chaos. The presence of horseshoe in any of the period 1, 2,…, n maps also indicates chaos. An illustration of the presence of horseshoe for a set of parameter values is given in Supplementary Section 2. The intersection of the ${\tilde{f}}_{\omega }^{1}$, ${\tilde{f}}_{\omega }^{2}$, ${\tilde{f}}_{\omega }^{3}$ maps with *ω*_*n*_ = *ω*_*n*+1_ indicate period 1, 2, 3 orbits, respectively. Figures 10–12B illustrate how the maps change with respect to the change of parameters.

Variation of the parameter τ_*l*_ or the magnitude of premature activation (as ϕ is set to -1.57 rad.) results in a set of rich dynamic behaviors as shown in Figure 10. The presence of the periodic orbits starts appearing approximately around τ_*l*_ ≈ 3 N m, where, the maps tangentially intersect the *ω*_*n*_ = *ω*_*n*+1_ line. The intermittency thus generated could elicit a period of slow walking (as *ω*_*n*_ and *ω*_*n*+1_ are less than -0.5 rad. s^−1^) as observed in PD. The period 3 orbits are generated upon a further increase in τ_*l*_. As can be seen from the maps in Figures 10–12B, a higher initial value of *ω*_*n*_ (e.g., *ω*_*n*_ > 0.45 rad. s^−1^ for τ_*l*_ ≈ 3 N m) results in a further increase in *ω*_*n*+1_ and gets attracted toward the periodic orbit of higher absolute value of angular velocity. This explains how swaying back and forth helps the PD patients in getting out of a freeze. Increasing τ_*r*_ results in almost opposite behavior as that of τ_*l*_ (Figure 12A). Varying ϕ can result in chaotic behavior as shown in Figure 9, and, Figure 11 indicates the variation in the maps which leads to this behavior. The neural control of the activity of plantar flexors is not explicitly modeled here. However, coming out of freeze could be the result of an increase of τ_*r*_ or decrease τ_*l*_ or increased initial absolute angular velocity generated by swaying. A low absolute value of angular velocity (voluntary or involuntary) or decrease of τ_*r*_ or increase of τ_*l*_ results in freezing (angular velocity moving to the region where *ω*_*n*_ = 0 rad. s^−1^). This explains the higher chances of freezing episodes even when the subject reduces the velocity (voluntarily/involuntarily) near narrow passages. Increase in the step length or |*θ*_*reset*_| results in freezing at comparatively higher absolute angular velocities (Figure 12B). But it may be noted that, typically, an increased step length is also associated with an increased absolute angular velocity due to inertia and therefore could be beneficial. There is likely an optimum step length for every subject as there is a trade-off between fatigue and initial angular velocity, which warrants further study.

### 4.4. Bifurcations of the One Dimensional System for the Inverted Pendulum Model

**Figure 14:**
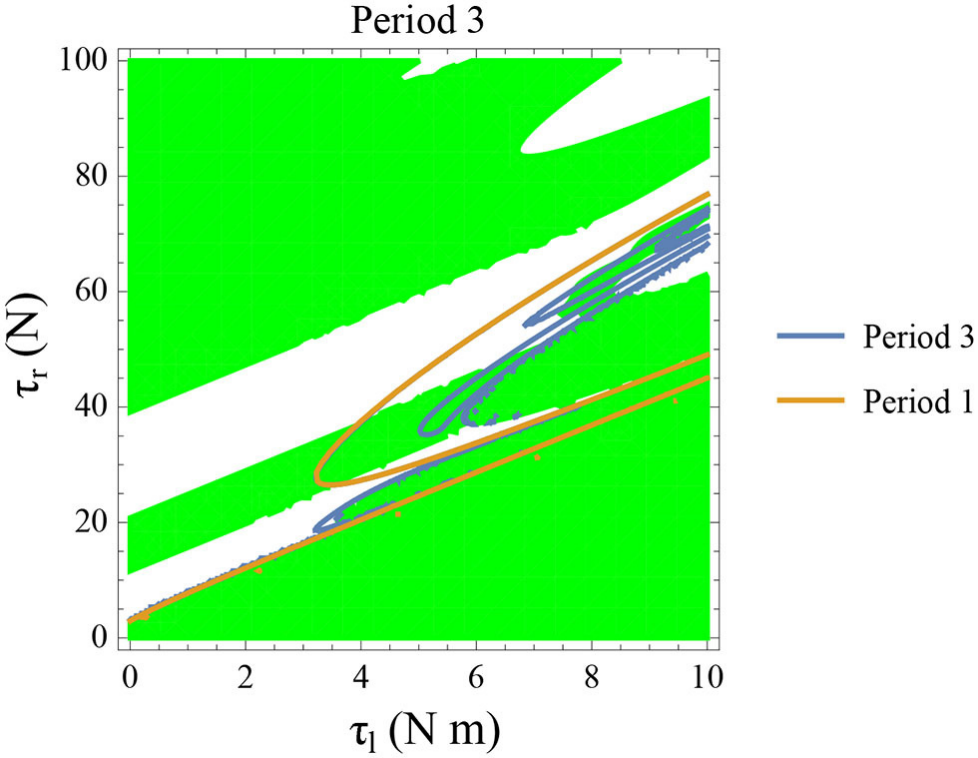
Constant parameters used are ϕ = −π/2 rad., *ω*_0_ = −0.4 rad. s^−1^, and *θ*_*reset*_ = −0.1 rad. The stable period 3 region is shown in green. The intersection of the period 3 orbits (in blue) and the stable regions form the region of stable period three orbits. The presence of period 3 orbits implies orbits of all other periods and therefore chaos.

Even though for most of the regions, the slope of the map in relation to the *ω*_*n*_ = *ω*_*n*+1_ can be identified visually, the stability of the system is not explicitly studied in the previous section. The contour of ${\tilde{f}}_{\omega }^{n}\left(x,\;\tau_{l},\;\tau_{r},\phi \right)=x$ for n=1 and 3 are plotted for variation in parameters in Figures 13, 14, respectively. The stability is computed by taking the derivatives (numerically) for the maps described in Figures 10–12B. These contours show how the points of intersection with *ω*_*n*_ = *ω*_*n*+1_ line for the maps shown in Figures 10–12B change upon variation in parameters.

Period one orbits are the normal walking cycles. The existence of these orbits in both low and high angular velocity conditions and different parameter variations are shown in Figure 13. In Figure 13A, two fixed points comes closer to each other and completely vanish for high values of τ_*l*_ resulting in a complete lack of periodic solutions. Typically walking could be ascribed to the stable region for periodic orbits, but, when *ω*_0_ is lower, and τ_*l*_ is non-zero, another periodic point emerges in the low-velocity regimes. This, therefore, results in slow-walking regions which under perturbations could lead to freezing. Also, at low-velocity regimes, the region is discontinuous and unstable for small perturbations of the parameter values or initial conditions. The stable periodic orbit moves to lower absolute value of angular velocities as τ_*l*_ is increased and eventually disappears.

The behavior observed while decreasing τ_*r*_ is analogous to increase in τ_*l*_. Figure 13B illustrates how changing τ_*r*_ and *ω*_0_ results in creation/destruction of the periodic orbits. It can be seen that at a sufficiently low value of τ_*r*_ the periodic orbit disappears. A higher value of τ_*r*_ results in the separation of the periodic orbits resulting in higher stable walking angular speeds. A similar behavior could be observed while decreasing τ_*l*_ in Figure 13A.

Initial angular velocity plays a major role in the behavior of the system. The effect of neural control parameters τ_*l*_ and τ_*r*_ in generating periodic behavior has been illustrated for lower and higher absolute angular velocity conditions in Figures 13C,D, respectively. In Figure 13C, the periodic orbit appears stable only for a tiny fraction of the parameters space. This is due to the highly discontinuous map shown previously. Conversely, at higher initial angular speeds the period one orbit is stable as shown in Figure 13D. It can be seen that an increase in τ_*l*_ moves the periodic orbit into an unstable region resulting in the possibility of a freeze. The presence of these orbits could only be seen in the low-velocity regions of the maps. Orbits of minimal period three indicate chaos and the presence of every other periodic orbits (Glendinning, 1994). The period 3 orbits for the variation of the parameters τ_*l*_ and τ_*r*_ is shown in the Figure 14. The Period 3 orbit is shown in blue and the period one in yellow.

## 5. Discussion, Summary, and Future Work

Freezing of gait results from a complex set of interacting physiological systems which consist of the brain, spinal cord, musculoskeletal system and external disturbances (Nutt et al., 2011). The model explains how a lack of coordination between central pattern generators of the plantar flexors of the leading leg and trailing leg (Nieuwboer et al., 2004) could lead to freezing and variability of walking.

A model of the torques generated by the plantar flexors acting on the stance leg has been proposed, and its effect on a biped and a reduced inverted pendulum model has been studied. The pattern of freezing observed in the model matches well with the behavior observed experimentally^7^ in Nutt et al. (2011) and Figure 5A). The equilibrium point description (Feldman, 1986; Sainburg, 2015) of the control of the muscles is avoided here and instead, we opted for an explicit control signal. However, variabilities in the “torque-length-characteristics” (Feldman, 1986) for a particular (set of) equilibrium point (points) can generate torques required for motion. Therefore, a parallel between the equilibrium point hypothesis of postural balance and our model can be drawn if the torques prescribed in the model are assumed to be the result of variabilities in the “torque-length-characteristics.”

Chaotic regions are observed to be closer to those regions where freezing ensues. In the inverted pendulum model, these regions show up only at low absolute angular velocity initial conditions. This may explain why freezing is a “rarely” occurring intermittent condition. This also may explain why freezing happens near obstacles or narrow paths where the subject voluntarily slows down to reduce the probability of collision. Obstacles could be either perceived or real. Hence, even though the pattern of freeze remains the same the causes could be varied. It might even be possible that the control of τ_*l*_ is driven by perceived obstacles or anxiety about the consequence of freezing (Ehgoetz Martens et al., 2014; Martens et al., 2016). Increase in τ_*l*_, therefore, could be thought to be indirectly influenced by anxiety and perceived obstacles. However, this hypothesis warrants further experimentation.

Varying the parameter step length which controls the stride length is observed to affect the maps and therefore the freezing regions. The results indicated that keeping the steps closer to each other such that |*θ*_*reset*_| is minimized, is safer for the PD patient. The stability of the period 2 & 3 orbits are highly sensitive to small variations of parameters (ϕ, τ_*r*_, τ_*l*_) which are proposed to be the reason for sporadic variabilities in gait seen in PD subjects. We also hypothesize that stable low absolute angular velocity regions of the state space for some parameter values form a “cantor set” and necessitates further study.

It can be speculated that a reason for the observed help of auditory/sensory cues (Rochester et al., 2005; Young et al., 2014; Amini et al., 2019) in reducing instances of freezing, is by indirectly forcing PD patients to make shorter steps with lesser variability, thus reducing the possibility of moving into the freezing region of walking. Variability in the walking times observed in the Inverted pendulum model translates to variability in step lengths in the biped model. Biped model shows a more complicated dependence on the parameters to eventual freezing (Figure 6A). This dependence is also a function of the initial conditions and could be investigated further in future work along with detailed bifurcation analysis.

The CPG activity is controlled by feedback mechanisms with delays, noise and input from the brain (which in turn is affected by different factors, including emotional state). The ground and other environmental conditions also play a role in walking. These variabilities are not accounted for in our model, which represents a limitation of the study. Like any other studies which are based on a mathematical model and numerical simulations, our results and conclusions also might not necessarily represent the entire spectrum of patients. Further extensive patient-based studies are to be performed prior to use of these ideas for the treatment of PD gait. As future work, a more detailed model is planned to include these variabilities. The future models will be compared with the simpler versions to understand the minimum set of variables generating the abnormal walking behavior. The key aspects explained using the proposed model can be summarized as follows

1. The higher variability in PD patients could be the result of parameters being closer to the point of chaos. A further change of the parameters can result in freezing. Therefore, increased variability should be looked at with caution (clinically) and should be treated to reduce it. The difficulty in the prediction of freezing also owes to the horseshoe near the freezing regions.
2. The pattern of reducing the step-sizes before freezing has been shown to be the result of slowing down (Figure 5A). Voluntary/involuntary reduction in angular velocity (in absolute terms) near the obstacles makes the subject more susceptible to freezing and highly irregular walking.
3. One plausible reason why sensory cues such as auditory or visual cues help in freezing is by reducing step lengths. The proposed model shows that the reduction in step length helps in reducing freezing episodes at lower absolute value of angular velocity conditions as it moves the patient away from the freezing region. Further experimental study is needed to understand the clinical applicability.

## Data Availability Statement

The raw data supporting the conclusions of this article will be made available by the authors, without undue reservation, to any qualified researcher.

## Author Contributions

MP and PM have contributed in design, implementation, analysis and editing of the study and manuscript. KT-A has contributed to the design and analysis of the study and editing of the manuscript. MW has contributed to the design of the study and editing of the manuscript. All authors contributed to the article and approved the submitted version.

## Conflict of Interest

The authors declare that the research was conducted in the absence of any commercial or financial relationships that could be construed as a potential conflict of interest.

## Footnotes

^1^Here *a* ↦ *b* indicates state *a* “maps to” state *b* after some time *t* where *t* ≥ 0,

^2^${\left[x_{1}^{-},x_{2}^{-}\right]}^{T}$ and ${\left[x_{1},x_{2}\right]}^{T-}$ are used interchangeably, where, *x*_1_ and *x*_2_ are components of some vector

^3^*p*^−^ and *p*^+^ refers to any parameter *p* before and after collision, respectively, in a particular step cycle.

^4^θ(*t*) and ω(*t*) are multi-valued functions at the point of collision.

^5^Even though the symbol θ(*T*) is used at the point of collision, it may be noted that this is a one to many mapping and therefore is not a single valued function in the traditional sense of the word.

^6^The solution to this optimization problem may not always exist.

^7^Here we are referring to the Figure 1 in Nutt et al. (2011). Source of the figure :https://pubmed.ncbi.nlm.nih.gov/21777828/#&gid=article-figures&pid=figure-1-uid-0
