# Supplementary material for: Ankle Push-Off Based Mathematical Model for Freezing of Gait in Parkinson's Disease
Source: Front Bioeng Biotechnol. 2020 Oct 29;8:552635. doi: 10.3389/fbioe.2020.552635 (PMC7658398; doi:10.3389/fbioe.2020.552635)
Supplement: Supplementary file 1 [file Data_Sheet_1.PDF]

Supplementary Material

1 TERMS OF THE ANALYTICAL SOLUTION

The terms N_1, N_2, D_1, D_2 of the analytical solution are given below.

$$\begin{aligned} N_1 = l(\Psi_N^2 + \Omega_1^2)(lm(\Psi_N^2 + \Omega_2^2)(\theta_0\sqrt{l}\Psi_N^2(e^{2t\Psi_N} + 1) + \omega_0\sqrt{l\Psi_N^2}(e^{2t\Psi_N} - 1)) \\ - \tau_r \sin(\theta_h)(\Omega_2(e^{t\Psi_N} - 1)(\sqrt{l}\Omega_2(e^{t\Psi_N} - 1) + \sqrt{l\Psi_N^2}(e^{t\Psi_N} + 1)) + \\ \sqrt{l}\Psi_N^2((e^{t\Psi_N} - 1)^2 - 2e^{t\Psi_N}\sin(t\Omega_2)))) \\ + \tau_l(\Psi_N^2 + \Omega_2^2) \\ (\sqrt{l}\Psi_N^2((e^{t\Psi_N} - 1)^2 - 2e^{t\Psi_N}\sin(t\Omega_1 + \phi) + \sin(\phi)(e^{2t\Psi_N} + 1)) \\ + \Omega_1(e^{t\Psi_N} - 1)(\sqrt{l}\Omega_1(e^{t\Psi_N} - 1) + \cos(\phi)\sqrt{l\Psi_N^2}(e^{t\Psi_N} + 1))) \end{aligned} \tag{S1}$$

$$D_1 = 2l^{5/2}m\Psi_N^2 e^{t\Psi_N}\left(\Psi_N^2 + \Omega_1^2\right)\left(\Psi_N^2 + \Omega_2^2\right) \tag{S2}$$

$$\begin{aligned} N_2 =& l(\Psi_N^2 + \Omega_1^2) \\ & (lm(\Psi_N^2 + \Omega_2^2)(\theta_0\sqrt{l}\Psi_N^2(e^{2t\Psi_N} - 1) + \\ & \omega_0\sqrt{l\Psi_N^2}(e^{2t\Psi_N} + 1)) \\ & - \tau_r \sin(\theta_h)(\Omega_2(\sqrt{l}\Omega_2(e^{2t\Psi_N} - 1) + \\ & \sqrt{l\Psi_N^2}(e^{2t\Psi_N} + 1)) - 2\sqrt{l}\Omega_2\Psi_N e^{t\Psi_N}\cos(t\Omega_2) + \sqrt{l}\Psi_N^2(e^{2t\Psi_N} - 1))) \\ & + \tau_l(\Psi_N^2 + \Omega_2^2) \\ & (-2\sqrt{l}\Omega_1\Psi_N e^{t\Psi_N}\cos(t\Omega_1 + \phi) \\ & + \Omega_1(\sqrt{l}\Omega_1(e^{2t\Psi_N} - 1) + \cos(\phi)\sqrt{l\Psi_N^2}(e^{2t\Psi_N} + 1)) + \sqrt{l}\Psi_N^2(\sin(\phi) + 1)(e^{2t\Psi_N} - 1)) \end{aligned} \tag{S3}$$

$$D_2 = 2l^{5/2}m\Psi_N e^{t\Psi_N}\left(\Psi_N^2 + \Omega_1^2\right)\left(\Psi_N^2 + \Omega_2^2\right) \tag{S4}$$

and

$$\begin{aligned} \Omega_1 &= 2\pi f r_1 \\ \Omega_2 &= 2\pi f r_2 \\ \Psi_N &= \sqrt{g/l} = \text{ reciprocal of the natural frequency} \end{aligned} \tag{S5}$$

2 GRAPHICAL ANALYSIS OF THE MAP BETWEEN STANCE PHASES

A horseshoe is a characteristic of a map which indicate chaos under iteration. Here, the presence of horseshoe has been shown for a set of parameter values in the physiological range for the map developed($\tilde{f}_\omega$). Using the conditions for horseshoe and therefore chaos, given in Glendinning (1994), the presence of the same could be shown for $\tilde{f}^n_\omega$.

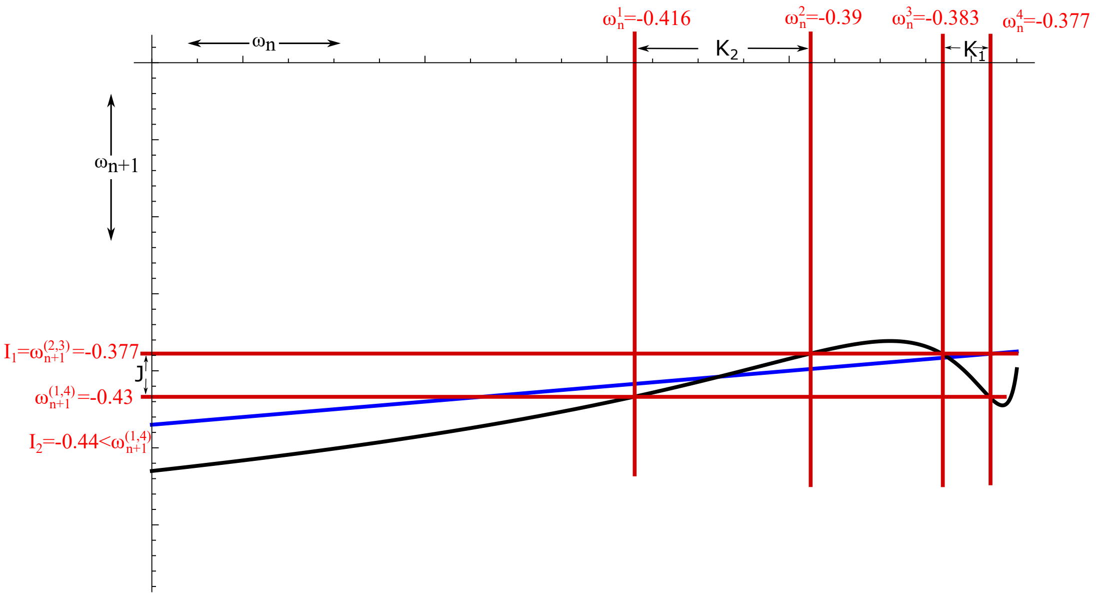

Figure S1: There is horseshoe in the interval between I_1 and I_2 for the map $\tilde{f}_\omega$. The proof is given in Sec.2. An unstable equilibrium is formed in the interval K2 ($|\dot{\tilde{f}}_\omega| > 1$) and an outward spiraling equilibrium in the interval K1($|\dot{\tilde{f}}_\omega| < -1$).

PROPOSITION 2.1. *For the map $\tilde{f}_\omega$ a horseshoe exists in the interval $I = [I_1, I_2] = [-0.377, -0.44]$.*

PROOF. Let $\omega_n^4 = -0.377, \omega_n^3 = -0.383, \omega_n^2 = -0.39, \omega_n^1 = -0.416, \omega_{n+1}^{(2,3)} = -0.377, \omega_{n+1}^{(1,4)} = -0.43, -0.44 = g < f$. Let the intervals be chosen in the following manner $I = [I_1, I_2], J = [\omega_{n+1}^{(2,3)}, \omega_{n+1}^{(1,4)}]$ and $\omega_n^4 = \omega_{n+1}^{(2,3)}$ (to avoid further confusion on the domain in which the function is defined). One also has, $k1 = [\omega_n^4, \omega_n^3], k2 = [\omega_n^2, \omega_n^1]$. $\tilde{f}_\omega(k2) \mapsto \tilde{f}_\omega(k1) \mapsto [\omega_{n+1}^{(2,3)}, \omega_{n+1}^{(1,4)}]$= epJ (end point preserved J). $K1 \subset J, K2 \subset J$ and $J \subset I$ and $\tilde{f}_\omega \in C^0(I, \mathbb{R})$. Therefore, there is a horseshoe in the interval.

REFERENCES

Glendinning, P. (1994). *Stability, instability and chaos: an introduction to the theory of nonlinear differential equations*, vol. 11 (Cambridge university press)
